# Isolation, Characterization, Genome Annotation, and Evaluation of Tyrosinase Inhibitory Activity in Secondary Metabolites of *Paenibacillus* sp. JNUCC32: A Comprehensive Analysis through Molecular Docking and Molecular Dynamics Simulation

**DOI:** 10.3390/ijms25042213

**Published:** 2024-02-12

**Authors:** Yang Xu, Xuhui Liang, Chang-Gu Hyun

**Affiliations:** Department of Beauty and Cosmetology, Jeju Inside Agency and Cosmetic Science Center, Jeju National University, Jeju 63243, Republic of Korea; iamxuyang1990@gmail.com (Y.X.); lxh03036@naver.com (X.L.)

**Keywords:** *Paenibacillus* sp., isolation, genome annotation, tyrosinase, molecular docking, molecular dynamics

## Abstract

A potential strain, *Paenibacillus* sp. JNUCC32, was isolated and subjected to whole-genome sequencing. Genome functional annotation revealed its active metabolic capabilities. This study aimed to investigate the pivotal secondary metabolites in the biological system. Fermentation and extraction were performed, resulting in the isolation of seven known compounds: tryptophol (**1**), 3-(4-hydroxyphenyl)propionic acid (**2**), ferulic acid (**3**), maculosin (**4**), brevianamide F (**5**), indole-3-acetic acid (**6**), and butyric acid (**7**). Tryptophol exhibited favorable pharmacokinetic properties and demonstrated certain tyrosinase inhibitory activity (IC_50_ = 999 μM). For further analysis of its inhibition mechanism through molecular docking and molecular dynamics (MD) simulation, tryptophol formed three hydrogen bonds and a pro-Michaelis complex with tyrosinase (binding energy = −5.3 kcal/mol). The MD simulation indicated favorable stability for the tryptophol–mushroom tyrosinase complex, primarily governed by hydrogen bond interactions. The crucial residues VAL-283 and HIS-263 in the docking were also validated. This study suggests tryptophol as a potential candidate for antibrowning agents and dermatological research.

## 1. Introduction

In 1991, the 16S rRNA gene sequences of standard strains representing 51 bacterial species were classified under the genus *Bacillus*, providing a more precise depiction of the phylogenetic relationships among these microorganisms [1,2]. Phylogenetic analysis revealed that the *Bacillus* sequences could be categorized into at least five distinct clusters. Subsequently, in 1993, Ash identified one of these clusters through an analysis of rRNA group 3 bacilli and designated it as *Paenibacillus* [3].

*Paenibacillus* emerged as a pivotal player in preventing crop diseases and promoting crop growth (indole-3-acetic acid, IAA) [4]. As research on *Bacillus* advances, various strains of *Paenibacillus* have been progressively isolated and characterized [5,6,7,8]. Whole-genome sequencing technology enables the comprehensive detection of all bacterial DNA, providing the complete gene sequence and unveiling the overall DNA composition of organisms [9,10]. Through the analysis of gene sequences and functional annotation, a more profound understanding of genetic variations both between and within species can be achieved [11]. These annotations include databases, such as COG (Clusters of Orthologous Groups), GO (Gene Ontology), KEGG (Kyoto Encyclopedia of Genes and Genomes), and others [12,13,14].

Certainly, we can not only explore the potential functional annotations of bacterial genomes at the genetic level but also study their biological activity in the secondary metabolites of *Paenibacillus*. Members of this genus can produce abundant secondary metabolites that have a wide range of activities (antibacterial, anti-inflammatory, inhibition of protease activity, phytohormones, etc.) [15,16,17,18].

Tyrosinase is widely distributed in animals, plants, and microorganisms, playing a crucial role in various processes, such as undesirable browning of food, skin pigment formation, antibiotic resistance, cuticle sclerotization, and neurodegeneration [19,20,21]. However, previous studies have shown that *Paenibacillus xylanilyticus* sp. and *Paenibacillus* sp. 1794 are negative for the production of tyrosinase [22,23]. Microorganisms from various genera have been found to possess antityrosinase activity. Kojic acid, as a well-studied tyrosinase inhibitor, was isolated from *A. candidus*, *A. albus*, and *A. niger* [24,25,26], and kojic acid dimethyl Ether and kojic acid monomethyl ether were identified from *Alternaria* sp. [27]. Seven isoflavones with antityrosinase activity were isolated from *Aspergillus oryzae* BCRC 32288 [28]. Byelyankacin isolated from *Enterobacterium* B20 has been shown to be a novel and potent inhibitor of melanogenesis [29]. Amphistin isolated from *Streptomyces* KP-3052 were shown to be a melanin production inhibitors [30]. *Lactobacillus helveticus*, as a probiotic, has produced a new tyrosinase inhibitor, which has been identified as cyclic tetrapeptide cyclo (-L-Pro-L-Tyr-L-Pro-L-Val-) [31].

Tyrosinase is a rate-limiting enzyme that plays an important role in the production of melanin. Generally, tyrosinase is mainly involved in the two-step reaction of melanin synthesis, including the hydroxylation of monophenols to o-diphenols and the oxidation of o-diphenols to o-quinones [32]. Tyrosinase is a tetramer (H2L2) copper-containing enzyme, and the crystal structure of the first full fungal tyrosinase complex is a 2.3A resolution structure (PDB ID: 2Y9W). Within this structure, CuA and CuB at the active center of the H subunit are hatched by histidine residues (His61, His85, His94, His259, His263, and His296) [33,34,35]. The binuclear copper binding site is at the bottom of a spacious cavity on the surface of the H subunit. This cavity is easily accessible and is not occluded by subunit side chain loops (H and L) [36,37].

Polyphenol oxidases (PPOs) are a family of isoenzymes widely distributed in a variety of organisms, including plants, bacteria, fungi, and animals [38]. Four tyrosinase genes (PPO1, PPO2, PPO3, and PPO4) have been identified in the common button mushroom *Agaricus bisporu* (AbTYR), and the PPO3 protein (PDB ID: 2Y9X) has been widely used in molecular docking studies of anti-browning agents and whitening agents [39,40,41,42]. The co-crystal ligand of mushroom tyrosinase is tropolone, which is a potent inhibitor of mushroom tyrosinase. Tropolone derivatives with a hydroxyl group at the 2-position are the precursors of many azulene derivatives (such as 2-methylazulene-1-carboxylate) [43,44,45]. The tropolone forms a pro-Michaelis complex with AbTYR [46]. Tropolone does not bind directly with copper ions, but rather near copper sites, and the molecule does not interact with H_2_O molecules in the binding site [47]. In previous studies, the results of molecular docking showed that there is a π interaction between H263 and tropolone in the mushroom tyrosinase–tropolone complex. Tropolone forms hydrogen bonds with N260 and Ser282, and its binding energy is -4.56 kcal/mol. In the mushroom tyrosinase–kojic acid complex, kojic acid establishes a hydrogen bond with M280 and forms π bonds with H263, resulting in a binding energy of -4.45 kcal/mol [48]. The two complexes mentioned above exhibit relatively stable RMSD values, suggesting intense binding and minimal structural changes [49]. Arbutin is a triterpenoid compound found in natural plants, and it is a known potent tyrosinase inhibitor. In the mushroom tyrosinase–arbutin complex, arbutin forms hydrogen bonds with N260, G281, and V283 and π bonds with H263 [50]. In the mushroom tyrosinase-ascorbic acid complex, ascorbic acid forms hydrogen bonds with N81, H85, and A323 [51]. Regarding the gallic acid–AbTYR complex, it was observed that the compound forms two hydrogen bonds with protein residues (Asn260 and His61) [52]. His61, Asn260, and Met280 are mainly responsible for hydrogen bonding interactions with L-arabinose [53]. Trilobatin interacted with the five amino acid residues, Ser282, Gly281, Arg268, Asn260, and His244, through hydrogen bonding [54]. Both 4-MTC and catechol substrate molecules form conventional hydrogen bonds with Asn260 [55]. The results show that in 1,2,4-triazole hydrazone derivatives, the phenyl group forms a π–π stacking interaction with His-263 residue, and the hydrazone group forms a hydrogen bond with the Gly-281 residue [56]. In the synthesized kojic acid derivatives, the ligand was found to form hydrogen bonds with N81 and ser282 and form a π interaction with H263 [57].

In summary, previous studies found that N81, M280, and N260 were proposed to play roles in the binding substrate, and H263 was observed to form a π interaction in mushroom tyrosinase [48,58,59]. Molecular dynamics simulation [60] can verify docking results and can be applied to the detection of drug molecule binding sites and their mechanisms, mechanism research on functional proteins, protein folding, etc. [61,62,63].

In this study, the gene functional annotation of the complete gene sequence of *Paenibacillus* sp. JNUCC32 was carried out. In order to further analyze the potential role of the strain JNUCC32, fermentation and extraction were performed, and valuable secondary metabolites were screened and evaluated in terms of tyrosinase inhibitory activity through enzyme experiments, molecular docking, and MD simulations.

## 2. Results and Discussion

### 2.1. General Features of Paenibacillus sp. JNUCC32 Genome

The genomic sequencing of *Paenibacillus* sp. JNUCC32 was conducted by employing an Illumina HiSeq sequencer. A total of 6,941,504 base pairs of clean sequences were generated, exhibiting a GC content of 51.27%. This genome contains 6334 protein-coding genes, 73 tRNAs, and 24 rRNA operons. No plasmids were detected in the genome. The assembled sequence has been deposited in the NCBI GenBank under the accession ID CP062260.

**Figure 1:**
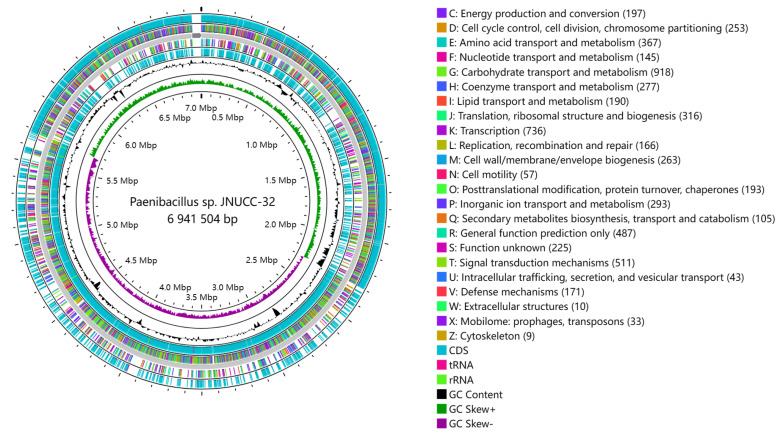
Circular map of the chromosome of Paenibacillus sp. JNUCC32.

A circular genome map provides a comprehensive visualization of various genome features, encompassing the distribution of genes on both positive and negative strands, and the functional classification of genes according to COG categories, GC content, genomic islands, and homologous genes. The genome atlas was drawn using CG View 1.0 (http://stothard.afns.ualberta.ca/cgview_server/, accessed on 20 October 2023) [64], as shown in Figure 1.

The innermost circle of the CG View circular plot (from inner to outer) signifies the genome size. The second circle represents the GC skew values, computed using the formula (G − C)/(G + C). Biologically, a positive value indicates a predisposition for transcription of coding sequences (CDSs) on the positive strand, while a negative value suggests a proclivity for CDS transcription on the negative strand. The third circle represents the G + C content of the genome. The fourth and seventh circles represent the presence of CDS, rRNA, and tRNA on the positive and negative strands, respectively. The fifth and sixth circles represent CDSs on the positive and negative strands, respectively. Different colors indicate distinct functional classifications based on the Clusters of Orthologous Groups (COG), with a total of 5049 genes annotated according to COG.

### 2.2. Genome Annotation

The gene sequences of the strain were compared with the COG, GO, and KEGG databases using Diamond, Blast, and Blast2GO v1.5.1 software, and functional annotations for the genome were obtained. Approximately 5049, 2084, and 2177 genes were annotated against the COG, GO, and KEGG databases, respectively.

#### 2.2.1. COG Database Annotations

The COG classification includes four categories: metabolism, information storage and processing, cellular processes and signaling, and poorly characterized proteins. The COG database categorizes protein functions into 26 classes, where each class is composed of orthologous sequences. The coding genes of strain JNUCC32 were classified into four categories within COG, comprising a total of 23 COG types. A total of 5049 gene functional annotations were obtained for the coding genes in the COG database, accounting for 81.50% of the CDSs.

**Figure 2:**
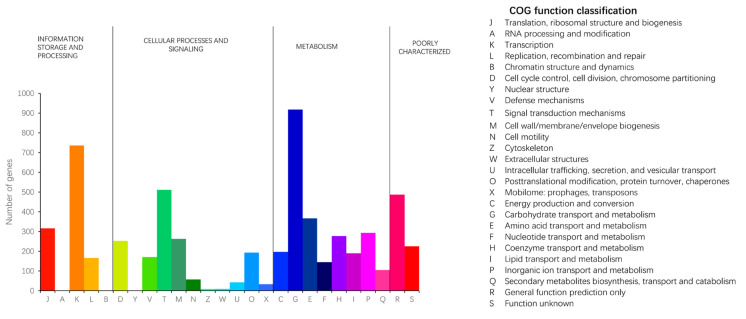
Functional classification of the genome against COG.

In strain JNUCC32, genes associated with carbohydrate transport and metabolism (COG category G) exhibited the highest annotation count (gene number: 918). Additionally, genes related to transcription (COG category K) showed a substantial annotation count (gene number: 736). Moreover, general functional prediction (COG category R) and proteins associated with amino acid transport and metabolism (category E) also received considerable gene annotations, indicating the strain’s pronounced ability to utilize carbohydrates and amino acids. The significant annotation count (gene number: 263) for the cell wall/membrane/envelope biogenesis category (COG category M) provides evidence supporting the inference that this strain may have robust capabilities in biofilm formation. Moreover, the annotation count (gene number: 171) for the defense mechanisms category (COG category V) indicates a potential resilience to external environmental factors. The results are shown in Figure 2.

#### 2.2.2. GO Database Annotations

The comparison of the genomic nucleotide sequence of strain JNUCC32 with the GO database protein sequences revealed the presence of genes associated with three major types: cellular components, biological processes, and molecular functions.

**Figure 3:**
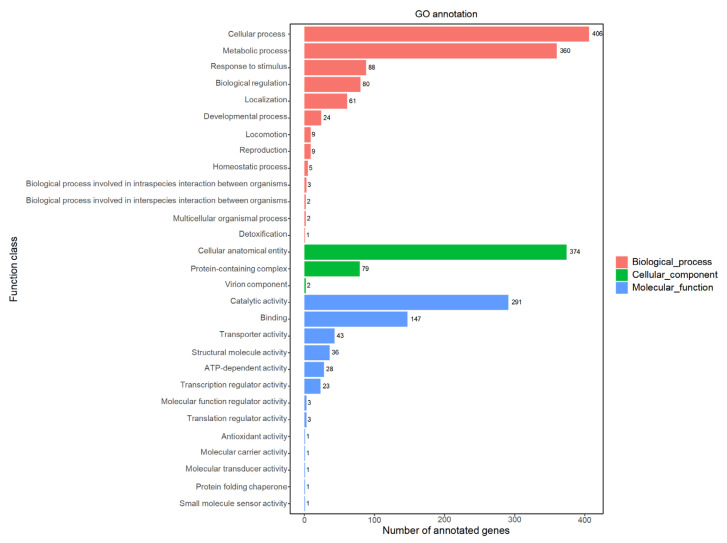
Functional classification of the genome against GO.

GO analysis suggested that biological-process-related genes (gene number: 2084) were the most abundant in strain JNUCC32, followed by genes related to molecular functions (gene number: 579) and cellular components (gene number: 455). Among the sub-functions annotated in the GO analysis, metabolic process (gene number: 360) and cellular process (gene number: 406) were dominant in the biological process category, while catalytic activity (gene number: 291) and binding (gene number: 147) were the core functions in the molecular function category. Cellular anatomical entity (gene number: 374) was dominant in the cellular component category. The results are shown in Figure 3.

#### 2.2.3. KEGG Database Annotations

The strain JNUCC32 has 2177 genes annotated in the KEGG database, with six primary functional categories: metabolism, genetic information processing, environmental information processing, cellular processes, organismal systems, and human diseases.

**Figure 4:**
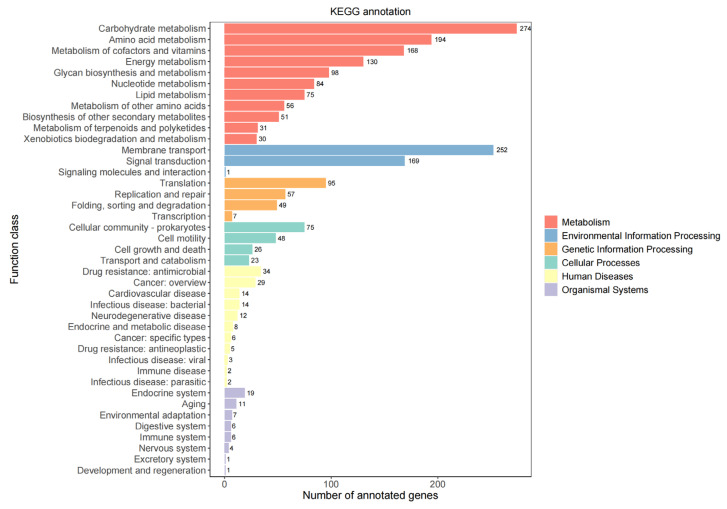
Functional classification of the genome against KEGG.

The annotation results included the total number of metabolic pathways and the number of genes involved in each metabolic pathway. It was found that more gene functional annotations were obtained at the level of metabolic pathways, especially carbohydrate metabolism, nucleotide metabolism, and amino acid metabolism. A total of 2177 genes were annotated in the metabolic pathways category. According to the KEGG database, the genome of strain JNUCC32 contains a variety of functional genes related to metabolism (gene number: 1191), including amino acid metabolism (gene number: 194), carbohydrate metabolism (gene number: 274), metabolism of cofactors and vitamins (gene number: 168), energy metabolism (gene number: 130), lipid metabolism (gene number: 75), and nucleotide metabolism (gene number: 84). A total of 422 genes were annotated in the environmental information processing category, with 252 genes related to membrane transport and 170 genes associated with signal transduction. The results are shown in Figure 4. However, the confirmation of gene expression requires further investigation and validation in subsequent studies.

### 2.3. Secondary Metabolites Isolated from Paenibacillus sp. JNUCC32

**Figure 5:**
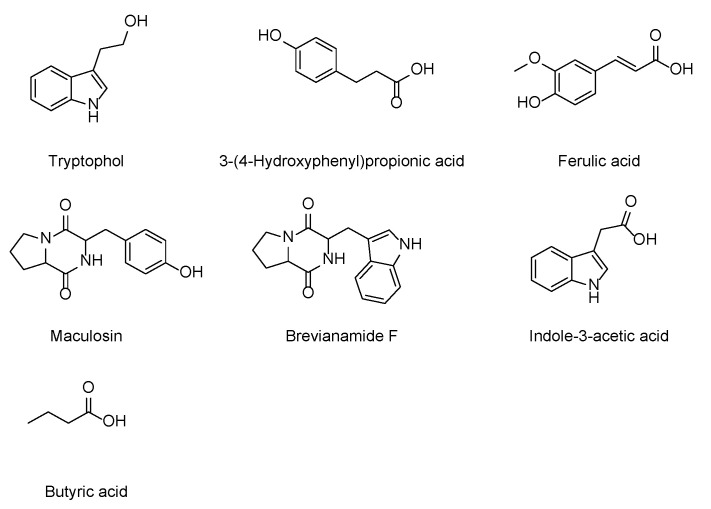
Chemical structure of the isolated compounds.

Fractionation and purification of the ethyl acetate extract (560 mg) of the culture broth of *Paenibacillus* sp. JNUCC32 led to the isolation of seven known secondary metabolites, namely tryptophol [65], 3-(4-hydroxyphenyl) propionic acid [66], ferulic acid [67], maculosin [68], brevianamide F [69], indole-3-acetic acid [70], and butyric acid [71], as shown in Figure 5.

### 2.4. Biological Activities of the Isolated Secondary Metabolites

**Figure 6:**
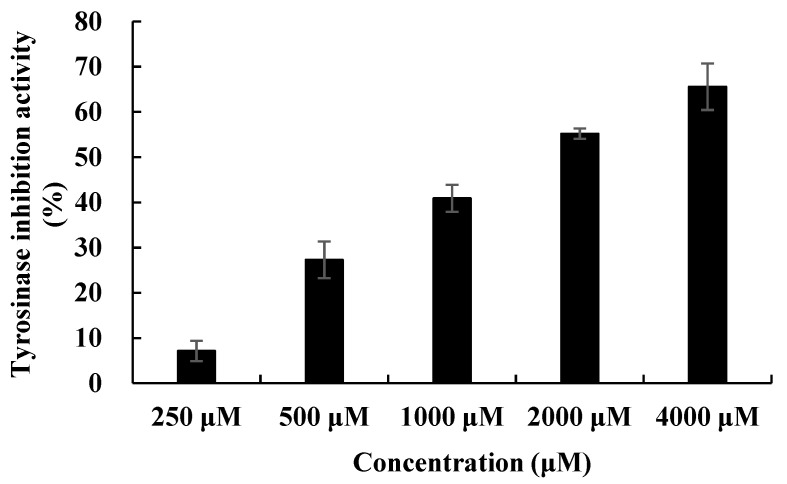
Tyrosinase inhibitory activity of tryptophol from *Paenibacillus* sp.

The tyrosinase activities of all compounds were screened. Among them, tryptophol showed tyrosinase activity with an IC_50_ value of 999 μM. Kojic acid and arbutin were used as positive controls, with IC_50_ values of 336.9 μM and 106.0 μm, respectively (Table 1, Figure 6).

### 2.5. Molecular Properties and Drug-Likeness

**Figure 7:**
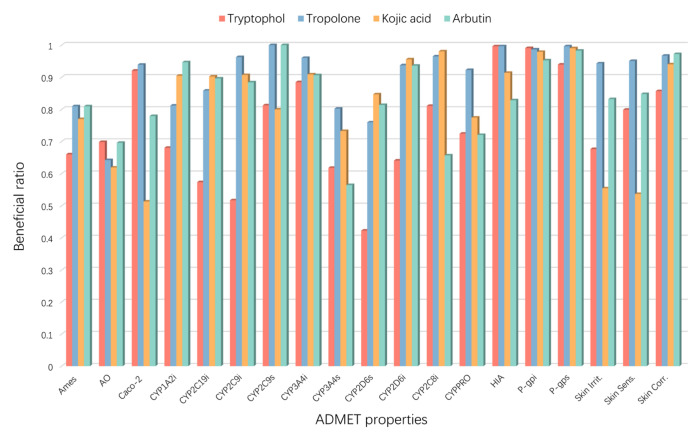
The ADMET properties of the compounds.

Drug candidates often undergo a significant attrition rate during discovery and development, primarily due to efficacy and safety, which are largely attributed to ADMET (absorption, distribution, metabolism, excretion, and toxicity) issues [72]. Using the web server admetSAR, 19 important ADMET properties (Figure 7, Table S1) were calculated, including Ames mutagenicity (Ames), acute oral toxicity (AO), Caco-2 permeability (Caco-2), CYP substrates and inhibitors (CYP1A2, CYP2C9, CYP2D6, CYP2C19, and CYP3A4), CYP inhibitory promiscuity (CYPPRO), human intestinal absorption (HIA), P-glycoprotein substrate (P-gps), P-glycoprotein inhibitor (P-gpi), skin irritation, skin sensitization, and skin corrosion, as shown in Figure 7.

Mutagenicity is one of the most crucial end points of toxicity [73]. Tryptophol exhibited the lowest Ames mutagenicity compared to the reference compounds. All compounds demonstrated similar acute oral toxicity. Tryptophol and tropolone exhibited better membrane permeability (Caco-2) than the reference compounds kojic acid and arbutin (0.9205, 0.9389, 0.5133, and 0.7795, respectively).

The cytochrome P450 (CYP) enzyme family is the most crucial enzyme system catalyzing the phase 1 metabolism of drugs and other xenobiotics [74]. CYP1A2 is an important metabolizing enzyme in the liver [75]. CYP2C9, CYP2C19, and CYP2D6 are characterized by polymorphic drug-metabolizing enzymes [76,77]. Tryptophol showed the lowest inhibition in CYP1A2, CYP2C9, CYP2C19, and CYP2D6 compared with other reference compounds.

Human Intestinal Absorption (HIA) scores provide qualitative gastrointestinal (GI) absorption descriptors. Tryptophol and tropolone showed that more than 99% of intestinal absorption far surpasses the minimal absorption criterion of 30% [78]. Tryptophol performed better than tropolone in skin irritation, skin sensitization, and skin corrosion.

In summary, the results indicate that the compound tryptophol has certain advantages in ADMET properties compared with the reference compounds.

The drug-likeness properties of the compounds were obtained through ADMETlab 2.0 SwissADME web server. Lipinski’s (Pfizer) Rule of Five (RO5) analysis evaluates the potential pharmacological properties of molecules during drug design [79]. With a molecular weight of 161.2 g/mol, a log *p* of 1.66, two hydrogen-bond donors, and two hydrogen-bond acceptors, tryptophol showed zero violations of Lipinski’s rule, demonstrating drug-like properties, as shown in Table 2.

The Ghose Filter, Veber’s rule, and the Egan rule are used to evaluate the drug-like properties of compounds. The Ghose Filter includes log *p* values (−0.4 to 5.6), molar refractivity values (40–130), molecular weight (160–480), number of atoms (20–70), and topological polar surface area (TPSA) ≤ 140 Å^2^ [80]. Veber’s rule states that the investigated compound has no more than 140 Å^2^ TPSA and 10 rotatable bonds [81]. The Egan rule states that a candidate drug has good oral absorption if −1.0 ≤ log *p* ≤ 5.8 and TPSA ≤ 130 Å^2^ [82].

The results show that only tryptophol complies with the Ghose Filter, and all compounds conform to Veber’s rule, the Egan rule, and Lipinski’s rule. Overall, tryptophol has better drug-like properties compared to other reference compounds.

### 2.6. Docking and Molecular Dynamics (MD) Simulations

#### 2.6.1. Molecular Docking

Typically, a binding energy less than 0 kcal/mol indicates spontaneous binding between the receptor and the ligand without the need for external energy. When the binding energy is below −5 kcal/mol, it signifies an excellent binding affinity. In the current docking study, the binding energy between the PPO3 protein and tryptophol was determined to be −5.3 kcal/mol, falling below the −5 kcal/mol threshold, indicating a high degree of binding between the PPO3 protein and tryptophol.

**Figure 8:**
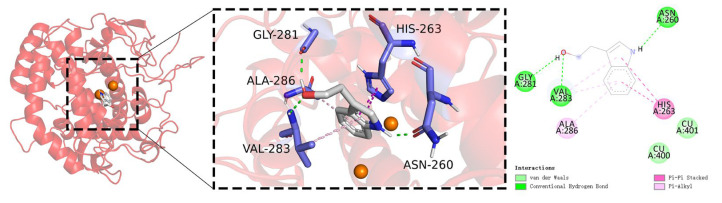
Molecular docking diagrams with 2D and 3D plots of the tryptophol complex.

The molecular docking results were visually analyzed using PyMOL 2.3.0 software and Discovery Studio 2019, and the detailed interactions are illustrated in Figure 8 [83,84]. This visual representation enhances the understanding of the docking outcomes, providing a comprehensive view of the molecular interactions.

Tryptophol is represented in gray, while blue-purple indicates amino acid residues within the protein that interact with tryptophol. Orange spheres denote copper ions. Interaction types are delineated by colored dashed lines: green for conventional hydrogen bonds, pale pink for π–alkyl interactions, light green for van der Waals forces, and purple for π–π stacking.

In the docking results between the PPO3 protein and tryptophol, hydroxyl and amino groups form three conventional hydrogen bonds with ASN-260, GLY-281, and VAL-283 and three π–alkyl interactions with VAL-283 and ALA-286, and the phenyl group forms a π–π stacking interaction with HIS-263 and two van der Waals interactions with two copper ions.

The visual analysis indicates that the PPO3 protein and tryptophol interact through a synergistic hydrophobic–hydrophilic mechanism. The docking binding energy of −5.3 kcal/mol suggests a strong interaction between the PPO3 protein and tryptophol, potentially influencing the structural functionality and biological activity of PPO3.

#### 2.6.2. Molecular Dynamics (MD) Simulations

However, the semi-flexible molecular docking approach currently employed lacks consideration of the protein structure’s flexibility. In order to further validate the degree and stability of the binding between the compound and the protein, this study conducted a 100 ns MD simulation of the PPO3–tryptophol complex.

**Figure 9:**
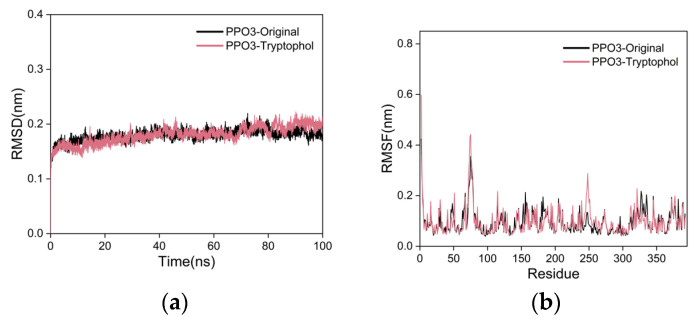
The results of MD simulations. (**a**) RMSD curves; (**b**) RMSF curves.

The RMSD curve serves as a key metric for assessing the stability of protein–ligand complexes [85]. A smaller RMSD value indicates minimal overall structural changes in the complex, signifying greater stability. In the RMSD curve for the PPO3 protein-tryptophol complex, although the tryptophol group is slightly less stable than the co-crystal ligand group, there are no significant fluctuations. The RMSD curve of co-crystallized ligand group almost overlaps with the curve of the tryptophol group. Therefore, RMSD analysis shows that the complex formed by PPO3 protein and tryptophol has better stability. The results are shown in Figure 9a. Validation of docking parameters of co-crystallized ligand and tryptophol, as shown in Figure S1.

The RMSF curve illustrates the extent of amino acid residue fluctuations within a protein during MD simulations [86]. Higher RMSF values indicate larger fluctuations, while lower values suggest minimal movement. The RMSF curve of the PPO3 protein–tryptophol complex demonstrates fluctuations within the 1 nm range, with no significant deviations. Notably, amino acid residues at positions 70–80 and 245–255, located at the periphery of the protein’s overall structure, exhibit reasonable fluctuations close to 0.5 nm. The RMSF curve of the co-crystal ligand group almost overlaps with the curve of the tryptophol group. These findings suggest that the addition of tryptophol has a minimal impact on the stability of amino acid residues in the PPO3 protein, indicating a high level of stability in the formed complex. The results are shown in Figure 9b.

**Figure 10:**
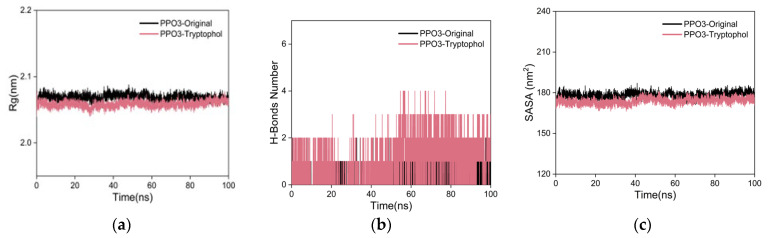
The results of MD simulations. (**a**) Rg curves; (**b**) H-bonds plot; (**c**) SASA plot.

The Rg is utilized to characterize the compactness and stability of a structure [87]. A larger Rg indicates a more pronounced expansion during MD simulations, while a smaller Rg suggests that the system remains compact and stable. As shown in Figure 10a, the Rg curve for the PPO3 protein–tryptophol complex exhibits fluctuations within the range of approximately 2.1 nm, maintaining overall stability without significant deviations. The Rg curve of the co-crystal ligand group almost overlaps with the curve of the tryptophol group. This result indicates the formation of a tight and stable complex between the PPO3 protein and tryptophol, with the addition of tryptophol not causing substantial alterations to the overall protein structure.

To investigate the hydrogen bonding properties at the binding site of the complex, this study calculated the number of hydrogen bonds involved in the stabilizing interactions between the ligand and the protein [88]. As shown in Figure 10b, during the 100 ns MD simulation, stable H-bond interactions did not form between the PPO3 protein and tryptophol. The H-bonds analysis exhibits significant fluctuations, indicating that the interaction between the PPO3 protein and tryptophol is primarily governed by a synergistic interplay between hydrophilic and hydrophobic interactions. The number of hydrogen bond ranges from 1–3, with a maximum of 4. The number of hydrogen bonds in the co-crystal ligand group was stable at one point during the simulation, indicating that a relatively stable hydrogen bond interaction was formed between the co-crystal ligand and the PPO3 protein, and the stability of the complex was slightly better than the tryptophol group.

The SASA is one of the factors used for studying protein folding and stability [89,90]. Proteins with stable structures often have more stable SASA curves. As shown in Figure 10c, the SASA curve of the PPO3 protein–tryptophol complex showed stable fluctuations throughout the entire process, without significant fluctuations, indicating that the complex formed by PPO3 protein and tryptophol has high stability, and the SASA curve of the co-crystal ligand group almost overlaps with the curve of the tryptophol group.

**Figure 11:**
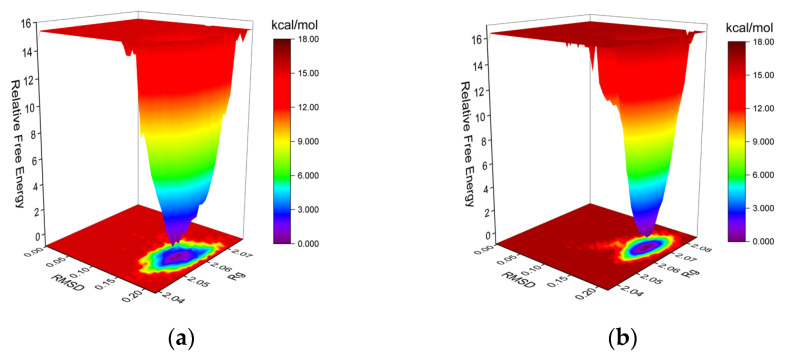
The plots of Gibbs FEL. (**a**) PPO3-Tryptophol; (**b**) PPO3-Original.

The Gibbs FEL was computed using the built-in Gromacs scripts g_sham and xpm2txt.py [91,92,93]. The Gibbs relative free energy was calculated based on the RMSD and Rg values, and the Gibbs FEL was plotted with the RMSD, Rg, and Gibbs relative free energy on the X, Y, and Z axes, respectively. The Gibbs FEL is used to describe the least-energy conformations in the whole process of dynamics simulation of complex structures. If the interaction between the protein and the ligand is weak or unstable, the Gibbs FEL may exhibit multiple rough clusters of minimum energy. Conversely, strong and stable interactions can form a single smooth energy cluster in the potential energy distribution. As shown in Figure 11, dark purple/blue spots represent the minimum energy values, indicating the most stable structures, while red/yellow spots represent unstable structures. The Gibbs FEL of both the co-crystal ligand group and the tryptophol group shows a single concentrated minimum energy cluster, and the energy cluster distribution is relatively concentrated, suggesting that the complex formed between the PPO3 protein and tryptophol is characterized by good stability.

**Figure 12:**
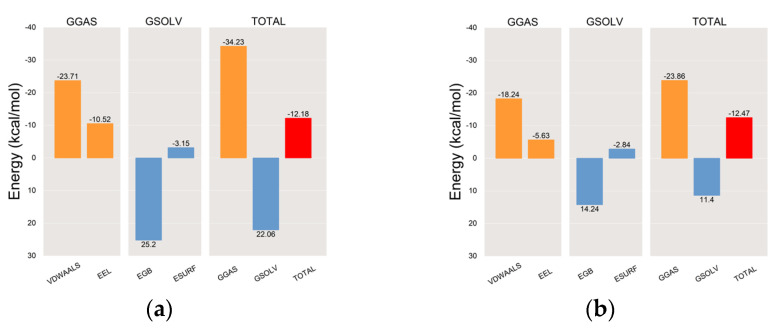
The plots of MM-PBSA binding energy. (**a**) PPO3-Tryptophol; (**b**) PPO3-Original.

Following the stabilization of the complex system, we computed the MM/PBSA binding energy for the protein–ligand complex [94,95]. The average binding free energy of PPO3 protein with the tryptophol group and the co-crystal ligand group were −12.18 kcal/mol and −12.47 kcal/mol, respectively, indicating a robust binding affinity between the PPO3 protein and tryptophol, as shown in Figure 12. VDWAALS, EEL, EGB, ESURF, GGAS, GSOLV, and TOTAL represent van der Waals forces, electrostatic energy, polar solvation energy, nonpolar solvation energy, molecular mechanics terms, solvation energy terms, and the average binding free energy, respectively.

**Figure 13:**
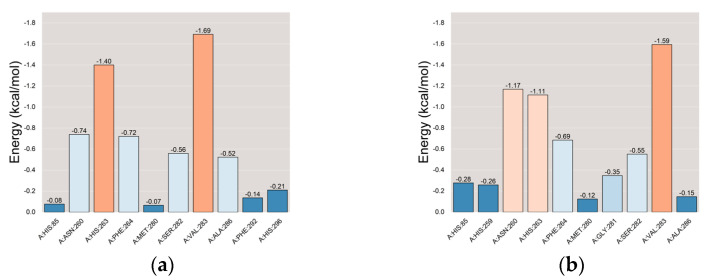
The plots of Residue-energy. (**a**) PPO3-Tryptophol; (**b**) PPO3-Original.

The results of Residue-energy [96] demonstrated that the amino acid residues VAL-283 and HIS-263 in the PPO3 protein exhibit the strongest binding affinity to tryptophol. This suggests a significant role for VAL-283 and HIS-263 in facilitating the binding of tryptophol to the PPO3 protein. The co-crystal ligand binds best to amino acid residues VAL-283, ASN-260, and HIS-263 residues in the PPO3 protein, indicating that VAL-283, ASN-260, and HIS-263 play a major role in the interaction between the co-crystal ligand and the PPO3 protein. The results are shown in Figure 13.

**Figure 14:**
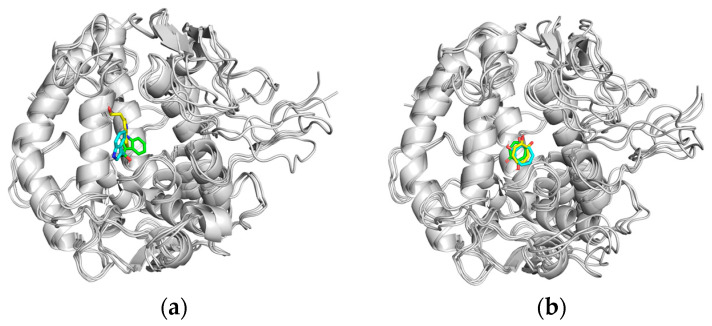
The binding mode of interaction in MD simulations. (**a**) PPO3-Tryptophol (**b**) PPO3-Original (yellow for 0 ns; green for 50 ns; blue for 100 ns).

To further investigate the binding situation of the complex before and after MD simu-lation, the complex structures at the time of 0 ns, 50 ns and 100 ns in the MD simulation trajectory of tryptophol group and co-crystallized ligand group were extracted and com-pared. It can be seen that the binding position of tryptophol and co-crystallized ligand with PPO3 protein has changed greatly before and after MD simulation, and the formed complexes have good stability, as shown in Figure 14.

## 3. Materials and Methods

### 3.1. Bacterial Isolation

*Paenibacillus* sp. JNUCC-32 was isolated from Baengnokdam in the summit crater of Halla Mountain in September 2019. The isolation methods are covered as follows: soil samples (0.5 g) were mixed with 0.45 mL of 0.1% tris-buffer (*w*/*v*) and shaken at 180 rpm, 30 °C, for 1 h. Subsequently, 100 µL of the suspension was serially diluted (10^−5^ to 10^−9^) and spread onto MRS medium. For routine culture, strain JNUCC32 was grown aerobically on Luria–Bertani solid medium and in Luria–Bertani liquid broth for 1 day at 30 °C, with maintenance in a 20% (*v*/*v*) glycerol suspension at −80 °C.

### 3.2. Genomic Analysis of Strain JNUCC32

The QIAGEN genomic-tip Kit (Qiagen Inc., Shenzhen, China) was used to extract whole genomic DNA from solid colonies of strain JNUCC32, and the genome was sequenced using PacBio RSII and Illumina platforms at Macrogen, Inc. (Seoul, Republic of Korea). The presence of plasmids was identified using PlasmidFinder 2.1.

The Prokka 1.14.6 command-line software tool was used for the comprehensive annotation of the draft genome [97]. Using BLAST, BLAST2go and Diamond sequence alignment tools, the obtained genes were compared with the COG (https://www.ncbi.nlm.nih.gov/COG/, accessed on 15 December 2023), GO (https://geneontology.org/, accessed on 15 December 2023), and KEGG (https://www.kegg.jp/, accessed on 15 December 2023) databases to obtain functional annotation information. The E-value parameter for Diamond was set to 10^−5^. The results of the gene functional annotation and MD simulations were input into R-4.3.2 software for graphical representation.

### 3.3. General Experimental Procedures

Luria–Bertani broth and Lactobacilli MRS broth were used (Becton, Franklin Lakes, NJ, USA). Silica gel (Merck, Darmstadt, Germany) and Sephadex LH-20 gel (Sigma-Aldrich, St. Louis, MO, USA) were used. Thin-layer chromatography (TLC) (Merck, Darmstadt, Germany) was performed. Tyrosinase from mushroom (T3824-50kv) was used (Sigma-Aldrich, St. Louis, MO, USA). Nuclear magnetic resonance (NMR) spectra were measured using a JNM-ECX 400 (FT-NMR system, 400 MHz, JEOL Co., Akishima, Japan).

### 3.4. Fermentation, Extraction, and Isolation

The strain *Paenibacillus* sp. JNUCC32 was cultured in a 250 mL flask containing 125 mL of Luria–Bertani medium at 30 °C for 48 h in a shaking incubator. Afterwards, the culture was transferred to 4 × 5 L flasks containing 1 L of NB with an inoculum of 5% (*v*/*v*). The strain JNUCC32 was cultured in LB medium under aerobic conditions at 30 °C for 4 days. The culture medium was filtered through 300 mm filter paper (ADVANTEC, Tokyo, Japan).

The culture solution (5 L) of the strain JNUCC32 was extracted with ethyl acetate (4 L × 3 times). The ethyl acetate extract was concentrated under reduced pressure to obtain a residue (560 mg). The EtOAc soluble fraction was subjected to vacuum liquid chromatography (VLC) on silica gel using a step gradient (chloroform–methanol, 300 mL each) to provide 10 fractions (Fr. V1–V10).

Compound **1** (11.3 mg) was isolated from combined Fr. V5 using Sephadex LH-20 column chromatography (CC) with chloroform–methanol (50:1, *v*/*v*). Compounds **2** (9.3 mg) and **7** (21.0 mg) were isolated from combined Fr. V8 using silica gel column chromatography (CC) with chloroform–methanol (20:1, *v*/*v*). Compounds **3** (14.6 mg) and **4** (10 mg) were isolated from combined Fr. V9 using silica gel column chromatography (CC) with chloroform–methanol (50:1, *v*/*v*). Compounds **5** (7.8 mg) and **6** (15.0 mg) were obtained from Fr. V10 using silica gel column chromatography (CC) with chloroform–methanol (10:1, *v*/*v*).The nuclear magnetic resonance (NMR) data of the compounds, as shown in Figure S2–S15.

#### 3.4.1. Tryptophol

^1^H NMR (400 MHz, DMSO-*D6*) δ 10.82–10.76 (m, 1H, H-12), 7.51 (dq, *J* = 7.9, 1.0 Hz, 1H, H-4), 7.33 (dt, *J* = 8.1, 1.0 Hz, 1H, H-7), 7.16–7.11 (m, 1H, H-1), 7.05 (ddd, *J* = 8.2, 7.0, 1.2 Hz, 1H, H-6), 6.97 (ddd, *J* = 7.9, 7.0, 1.1 Hz, 1H, H-5), 4.64 (t, *J* = 5.3 Hz, 1H, OH), 3.70–3.61 (m, 2H, H-10), 2.85 (td, *J* = 7.5, 0.9 Hz, 2H, H-9). ^13^C NMR (100 MHz, DMSO-*D6*) δ 136.17 (C-8), 127.45 (C-3), 122.80 (C-1), 120.80 (C-6), 118.37 (C-4), 118.15 (C-5), 111.57 (C-2), 111.32 (C-7), 61.75 (C-10), 28.89 (C-9).

#### 3.4.2. 3-(4-Hydroxyphenyl)propionic Acid

^1^H NMR (400 MHz, DMSO-*D6*) δ 12.07 (s, 1H, COOH-10), 9.16 (s, 1H, OH-11), 7.04–6.96 (m, 2H, H-2 and H-6), 6.69–6.61 (m, 2H, H-3 and H-5), 2.69 (t, *J* = 7.6 Hz, 2H, H-7), 2.49 -2.40 (m, 2H, H-8). ^13^C NMR (100 MHz, DMSO-*D6*) δ 173.96 (C-9), 155.55 (C-4), 130.95 (C-1), 129.13 (C-2 and C-6), 115.07 (C-3 and C-5), 35.77 (C-8), 29.60 (C-7).

#### 3.4.3. Ferulic Acid

^1^H NMR (400 MHz, DMSO-*D6*) δ 12.14 (s, 1H, COOH), 9.56 (s, 1H, H-11), 7.49 (d, *J* = 15.9 Hz, 1H, H-7), 7.28 (d, *J* = 2.0 Hz, 1H, H-2), 7.08 (dd, *J* = 8.3, 2.0 Hz, 1H, H-5), 6.79 (d, *J* = 8.1 Hz, 1H, H-6), 6.37 (d, *J* = 15.9 Hz, 1H, H-8), 3.81 (s, 3H, OCH3-12). ^13^C NMR (100 MHz, DMSO-*D6*) δ 168.08 (C-9), 149.13 (C-3), 147.96 (C-4), 144.59 (C-7), 125.82 (C-1), 122.91 (C-6), 115.67 (C-5), 115.55 (C-8), 111.14 (C-2), 55.71 (C-12).

#### 3.4.4. Maculosin

^1^H NMR (400 MHz, CHLOROFORM-*D*) δ 7.10–7.00 (m, 2H, H-2′ and H-6′), 6.81–6.73 (m, 2H, H-3′ and H-5′), 6.06 (s, 1H, NH-8), 4.22 (ddd, *J* = 9.9, 4.1, 1.7 Hz, 1H, H-9), 4.14–4.04 (m, 1H, H-6), 3.69–3.51 (m, 2H, H-3), 3.50–3.40 (m, 1H, H-10), 2.78 (dd, *J* = 14.5, 9.8 Hz, 1H, H-10), 2.37–2.25 (m, 1H, H-5a), 2.05–1.96 (m, 1H, H-5b), 1.94–1.82 (m, 2H, H-4). ^13^C NMR (100 MHz, CHLOROFORM-*D*) δ 169.95 (C-7), 165.35 (C-1), 155.81 (C-4′), 130.49 (C-2′ and C-6′), 126.94 (C-1′), 116.22 (C-3′ and C-5′), 59.26 (C-6), 56.41 (C-9), 45.56 (C-3), 36.05 (C-10), 28.45 (C-5), 22.57 (C-4).

#### 3.4.5. Brevianamide F

^1^H NMR (400 MHz, CHLOROFORM-*D*) δ 8.34 (s, 1H, NH-1), 7.59 (d, *J* = 7.9 Hz, 1H, H-4), 7.39 (dt, *J* = 8.1, 0.9 Hz, 1H, H-7), 7.23 (ddd, *J* = 8.2, 7.1, 1.2 Hz, 1H, H-6), 7.14 (ddd, *J* = 8.0, 7.0, 1.0 Hz, 1H, H-5), 7.11–7.07 (m, 1H, H-2), 5.76 (s, 1H, NH-14), 4.37 (ddd, *J* = 10.8, 3.9, 1.6 Hz, 1H, H-11), 4.07 (ddd, *J* = 9.0, 6.9, 1.6 Hz, 1H, H-14), 3.76 (ddd, *J* = 15.1, 3.7, 1.0 Hz, 1H, H-10a), 3.66 (dt, *J* = 11.9, 7.9 Hz, 1H, H-19b), 3.58 (ddd, *J* = 11.7, 8.6, 3.2 Hz, 1H, H-19a), 2.97 (dd, *J* = 15.1, 10.8 Hz, 1H, H-10b), 2.38–2.27 (m, 1H, H-17a), 2.09–2.01 (m, 1H, H-17b), 2.01–1.96 (m, 1H, H-18a), 1.96–1.81 (m, 1H, H-18b). ^13^C NMR (101 MHz, CHLOROFORM-*D*) δ 169.50 (C-12), 165.69 (C-15), 136.83 (C-8), 126.86 (C-9), 123.48 (C-2), 122.91 (C-6), 120.13 (C-5), 118.64 (C-4), 111.71 (C-7), 110.05 (C-3), 59.37 (C-14), 54.71 (C-11), 45.55 (C-19), 28.43 (C-17), 26.97 (C-10), 22.74 (C-18).

#### 3.4.6. Indole-3-acetic Acid

^1^H NMR (400 MHz, METHANOL-D3) δ 7.54 (dt, *J* = 7.9, 1.0 Hz, 1H, H-4), 7.34 (dt, *J* = 8.1, 1.0 Hz, 1H, H-7), 7.15 (d, *J* = 1.0 Hz, 1H, H-2), 7.09 (ddd, *J* = 8.2, 7.0, 1.2 Hz, 1H, H-6), 7.01 (ddd, *J* = 8.0, 7.0, 1.1 Hz, 1H, H-5), 3.73 (d, *J* = 0.9 Hz, 2H, H-10). ^13^C NMR (100 MHz, METHANOL-*D3*) δ 176.51 (C-11), 138.00 (C-8), 128.66 (C-9), 124.61 (C-2), 122.42 (C-6), 119.80 (C-5), 119.43 (C-4), 112.22 (C-7), 108.89 (C-3), 31.95 (C-10).

#### 3.4.7. Butyric Acid

^1^H NMR (400 MHz, METHANOL-*D3*) δ 2.26 (t, *J* = 7.3 Hz, 2H, H-2), 1.62 (q, *J* = 7.4 Hz, 2H, H-3), 0.95 (t, *J* = 7.4 Hz, 3H, H-4). ^13^C NMR (100 MHz, METHANOL-*D3*) δ 177.58 (C-1), 36.83 (C-2), 19.42 (C-3), 13.95 (C-4).

### 3.5. Biological Activity of Secondary Metabolites

#### Enzymatic Assay

For the enzymatic assay, a reaction mixture was prepared using 0.1 M of potassium phosphate buffer (pH 6.8), 2 mM of L-tyrosine, and 2500 units of tyrosinase, and samples of various concentrations were obtained. Twenty-microliter aliquots of samples at varying concentrations were added to a 96-well plate. Subsequently, 130 μL of the substrate solution (comprising buffer and L-tyrosine) was added, followed by the addition of 5 μL of tyrosinase and 45 μL of buffer solution. The reaction mixture was incubated at 37 °C for 10 min. The absorbance was then measured at 490 nm. Arbutin and kojic acid served as the positive controls in this assay.

### 3.6. Molecular Properties and Drug-Likeness

Pharmacokinetic parameters were evaluated using ADMET and drug-likeness properties analysis. The drug-likeness properties of compounds were were thoroughly examined by employing several models, including ADMETlab 2.0 [98], SwissADME (http://www.swissadme.ch/; accessed on 27 January 2024) [99]. The ADMET properties of compounds were examined through admetSAR 2.0 web server [100].

### 3.7. Molecular Docking Simulation

The receptor protein PPO3 (PDB ID: 2Y9X) was obtained from the Protein Data Bank (PDB) (http://www.rcsb.org, accessed on 19 November 2023), providing the 3D structure file of the protein. PyMOL 2.3.0 software was used to inspect the protein structure for subsequent docking procedures. The 3D structure file of the ligand small molecule tryptophol (PubChem CID: 10685) was retrieved from the PubChem database (https://pubchem.ncbi.nlm.nih.gov, accessed on 19 November 2023). The obtained 3D structure file underwent optimization using the MMFF94 force field in OpenBabel 2.4.1 software to attain an energetically favorable conformation, resulting in the lowest energy state of the optimal molecular structure [101].

The protein was subjected to hydrogenation using AutoDock Tools 1.5.6, and the small molecule underwent hydrogenation as well as determination of rotatable bonds [102]. The processed structures were saved as pdbqt files. The molecular docking parameters were configured in the grid section, referencing the position of the co-crystallized ligand the PPO3 protein. The docking parameters were set as follows: center_x = −10.2, center_y = −30.3, center_z = −44.4, size_x = 20, size_y = 20, and size_z = 21. The semi-flexible docking mode was chosen, and the export results were set to 50, with a maximum of 3,000,000 samplings. The docking algorithm employed was the Lamarckian genetic algorithm. AutoGrid4 and AutoDock4 were executed for molecular docking, resulting in binding free energies and docking result files.

### 3.8. Molecular Dynamics (MD) Simulations

Using Gromacs 2022 software, MD simulations were performed on the PPO3–tryptophol complex. Charmm36 was selected as the protein force field, with Gaff2 as the ligand force field, and the TIP3P water model was employed to solvate the protein–ligand system. A periodic boundary box of 1.2 nm was established for the water box. Sodium and chloride ions were added to balance the system’s charge, aiming to replicate the conditions of real experimental environments. Prior to formal MD simulations, the complex underwent 50,000 steps of energy minimization using the conjugate gradient algorithm. Subsequently, the system was subjected to 100 ps of further equilibration in the canonical ensemble (NVT) and the isothermal–isobaric ensemble (NPT) at 310 K and 1 atm. Finally, a 100 ns MD simulation was conducted under normal temperature and pressure.

This study analyzed various parameters of the MD trajectory of the complex, including root mean square deviation (RMSD), root mean square fluctuation (RMSF), radius of gyration (Rg), hydrogen-bond analysis (H-bond), solvent accessible surface area (SASA), Gibbs free energy landscape (FEL), molecular mechanics–Poisson Boltzmann surface area (MM-PBSA) binding energy, and energy contributions from amino acids involved in binding (Residue-energy).

## 4. Conclusions

The genome functional annotation of *Paenibacillus* sp. JNUCC32 indicates active metabolic ability. To further investigate the crucial secondary metabolic molecules, the strain was cultured and isolated. Among the secondary metabolites, tryptophol exhibited certain tyrosinase inhibitory activity. Tryptophol demonstrated certain advantages in terms of ADMET properties and exhibited better drug-likeness properties compared to other reference compounds.

To analyze the inhibition mechanism, the active site of the co-crystallized ligand (tropolone) of PPO3 protein (PDB ID: 2Y9X) was selected as the docking active site for molecular docking simulations. Firstly, the molecular docking results demonstrated that tryptophol forms hydrogen bonds with ASN-260, GLY-281, and VAL-283, consistent with the binding pattern observed in the docking of the arbutin molecule [47]. Secondly, similar to tropolone, tryptophol forms weak van der Waals forces with copper ions and forms a pre-Michaelis complex with mushroom tyrosinase [45,46]. Thirdly, tryptophol forms a π–π stacking interaction with the PPO3 protein (HIS-263), consistent with the docking results observed for kojic acid, tropolone, and arbutin [47,49].

The verification of docking results through the MD simulation indicated that the tryptophol–PPO3 protein complex exhibits excellent stability in terms of the RMSD, RMSF, Rg, SASA, and Gibbs FEL. H-bond analysis indicated that the complex is stabilized through hydrogen bond interactions. The MM/PBSA binding energy result (−16.4 kcal/mol) suggests a strong binding affinity, and in the study of Residue-energy, it was found that VAL-283 and HIS-263 play a significant role in the binding of tryptophol to the PPO3 protein, which is consistent with the molecular docking results indicating that VAL-283 and HIS-263 are the main interactions involved in binding. This result shows that tryptophol has certain potential in food science and dermatological research.

As a precursor of the auxin, tryptophol not only possesses hypnotic effect [103] and reduces the production of Prostaglandin E2 (PGE2), TNFα, and IFNγ [104,105], but also inhibits the activity of butyrylcholinesterase [106] and the proliferation of leukemia U937 cells [107]. Moreover, this study demonstrates that tryptophol has potential in food science and dermatology research.

## Figures and Tables

**Table 1:** Tyrosinase activity of compounds isolated from *Paenibacillus* sp.

Compounds	Tyrosinase Activity IC_50_ (μM)
Tryptophol	999
Maculosin	-
Brevianamide F	-
Indole-3-acetic acid	-
Kojic acid	336.9
Arbutin	106.0

**Table 2:** The drug-likeness properties of compounds.

Compound	MW	HBA	HBD	RB	TPSA (Å^2^)	Log *p*	MR	RO5	Ghose Filter	Veber Rule	Egan Rule	Drug Likeness
Tryptophol	161.2	2	2	2	36.02	1.661	49.23	Yes	Yes	Yes	Yes	Yes
Tropolone	122.1	2	1	0	37.30	0.565	34.74	Yes	No	Yes	Yes	No
Kojic acid	142.1	4	2	1	70.67	−0.878	33.13	Yes	No	Yes	Yes	No
Arbutin	272.3	7	5	3	119.61	−0.884	62.61	Yes	No	Yes	Yes	No

MW: Molecular Weight; HBA: Num. H-Bond Acceptors; HBD: Num. H-Bond Donors; RB: Num. Rotatable Bonds; MR: Molar Refractivity.

## Data Availability

Data are contained within the article and Supplementary Materials .

## Supplementary Materials

The supporting information can be downloaded at: https://www.mdpi.com/article/10.3390/ijms25042213/s1.
