# Supplementary material for: Isolation, Characterization, Genome Annotation, and Evaluation of Tyrosinase Inhibitory Activity in Secondary Metabolites of Paenibacillus sp. JNUCC32: A Comprehensive Analysis through Molecular Docking and Molecular Dynamics Simulation
Source: Int J Mol Sci. 2024 Feb 12;25(4):2213. doi: 10.3390/ijms25042213 (PMC10889091; doi:10.3390/ijms25042213)
Supplement: Supplementary file 1 [file ijms-25-02213-s001.zip › ijms-2845288-supplementary.pdf]

Isolation, Characterization, Genome Annotation, and Evaluation of Tyrosinase Inhibitory Activity in Secondary Metabolites of *Paenibacillus* sp. JNUCC32: A Comprehensive Analysis through Molecular Docking and Molecular Dynamics Simulation

Yang Xu, Xuhui Liang and Chang-Gu Hyun *

Department of Beauty and Cosmetology, Jeju Inside Agency and Cosmetic Science Center, Jeju National University, Jeju 63243, Republic of Korea
* Correspondence: cghyun@jejunu.ac.kr; Tel.: +82-64-754-1803

Supplementary materials

^{1}H NMR, ^{13}C NMR, RMSD, ADMET

CONTENTS

Figure S1. Validation of docking parameters of co-crystallized ligand and tryptophol 1

Figure S2. ^{1}H NMR of compound 1 2

Figure S3. ^{13}C NMR of compound 1 3

Figure S4. ^{1}H NMR of compound 2 4

Figure S5. ^{13}C NMR of compound 2 5

Figure S6. ^{1}H NMR of compound 3 6

Figure S7. ^{13}C NMR of compound 3 7

Figure S8. ^{1}H NMR of compound 4 8

Figure S9. ^{13}C NMR of compound 4 9

Figure S10. ^{1}H NMR of compound 5 10

Figure S11. ^{13}C NMR of compound 5 11

Figure S12. ^{1}H NMR of compound 6 12

Figure S13. ^{13}C NMR of compound 6 13

Figure S14. ^{1}H NMR of compound 7 14

Figure S15. ^{13}C NMR of compound 7 15

Table S1. The ADMET properties of the compounds 16

Figure S1. Validation of docking parameters of co-crystallized ligand and tryptophol. Yellow (before docking), blue (after docking).

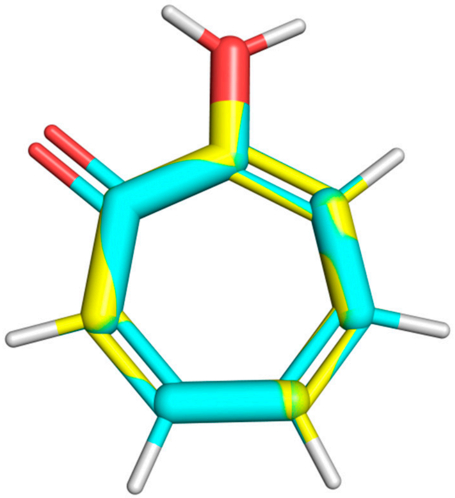	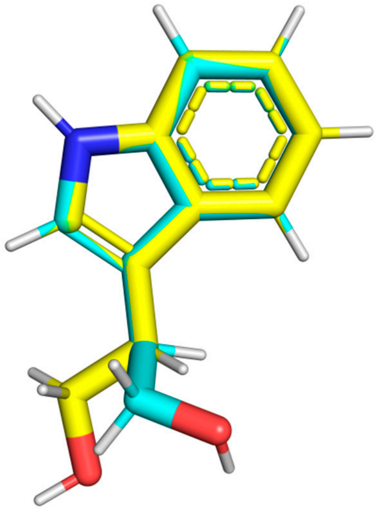
RMSD: 0.001Å	RMSD: 0.002Å
co-crystallized ligand	Tryptophol

Figure S2. ^{1}H NMR of compound **1**

Tryptophol

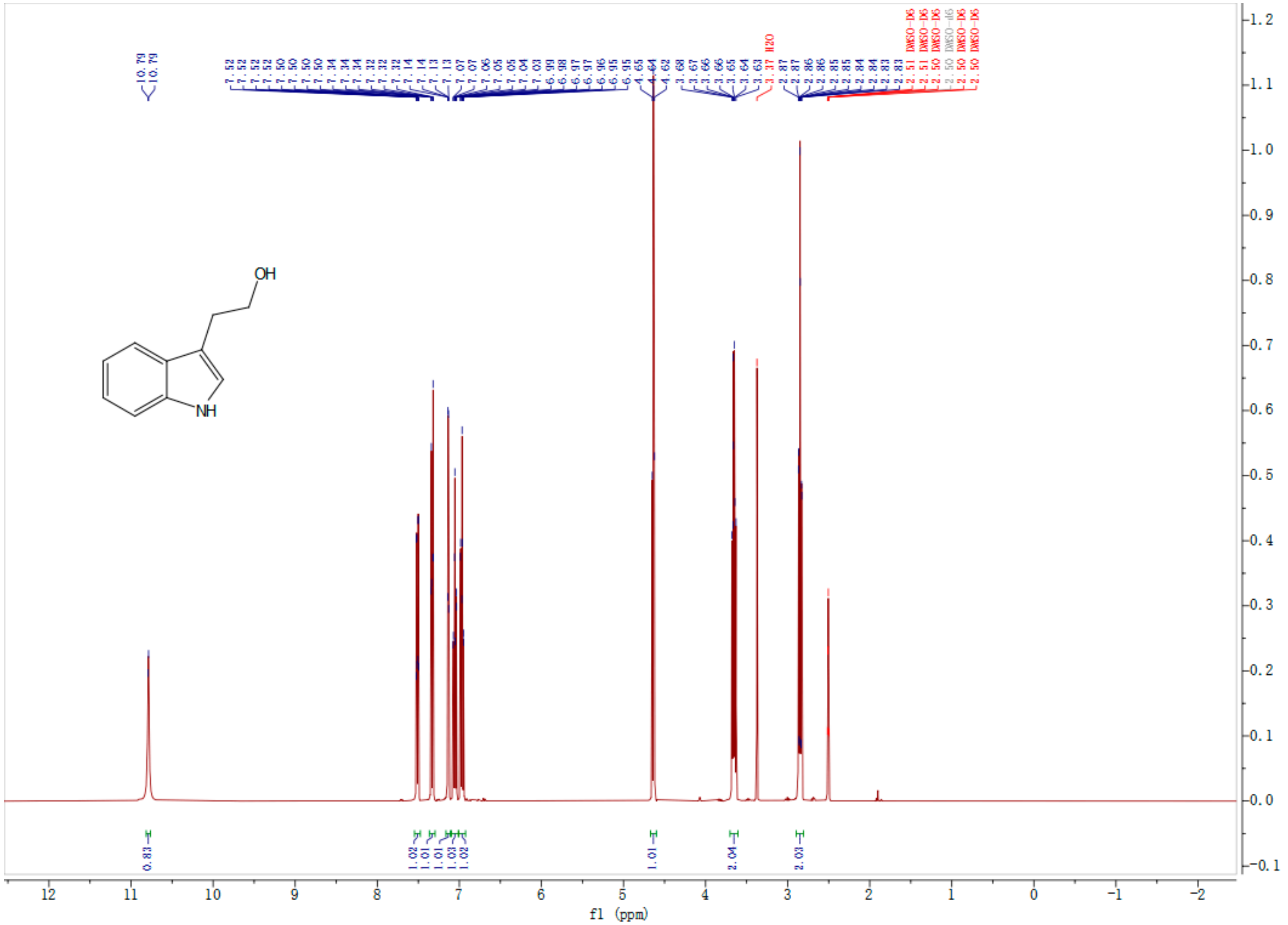

Figure S3. ^{13}C NMR of compound **1**

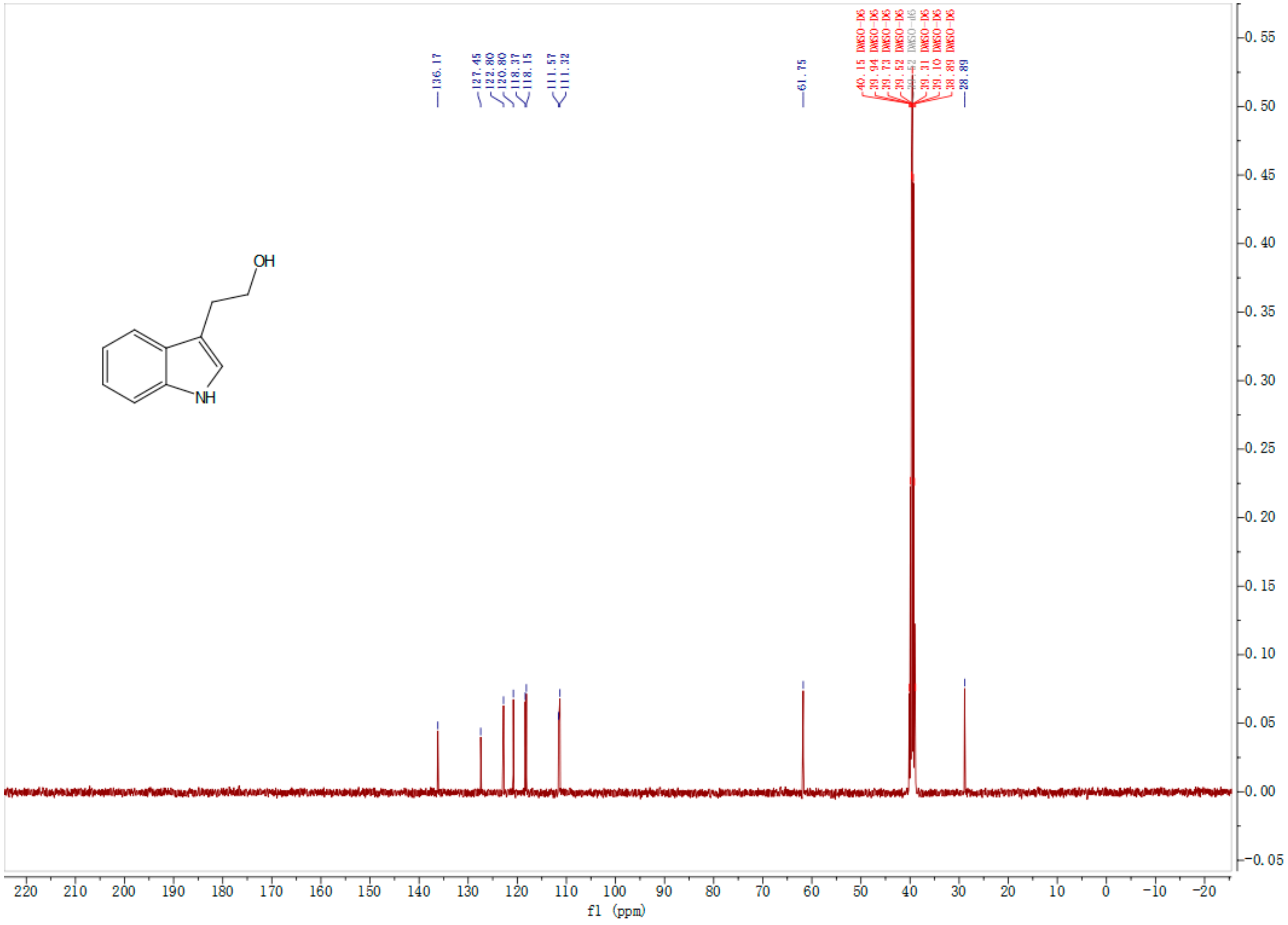

Figure S4. ^{1}H NMR of compound **2**

3-(4-Hydroxyphenyl)propionic acid

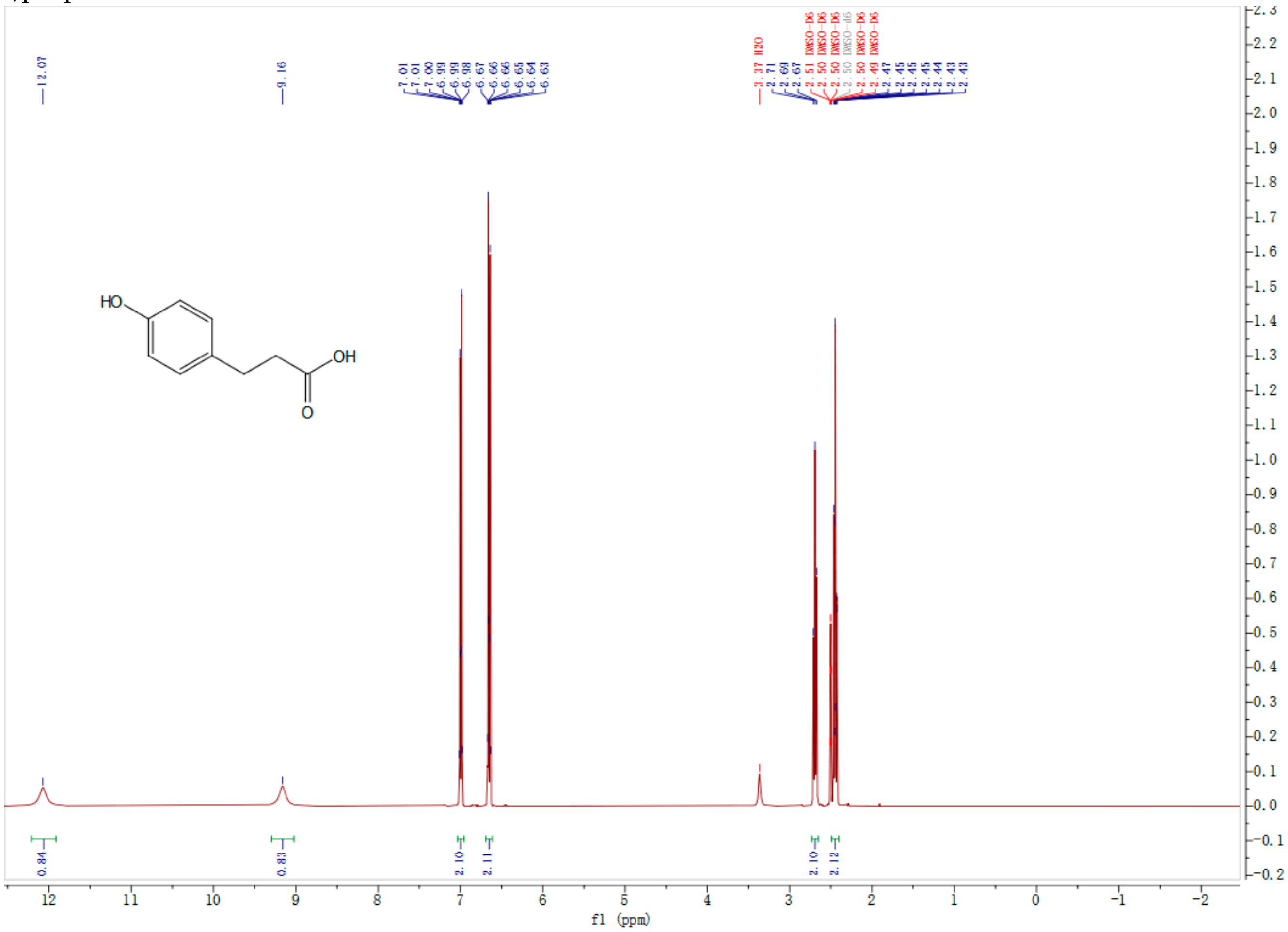

Figure S5. ^{13}C NMR of compound **2**

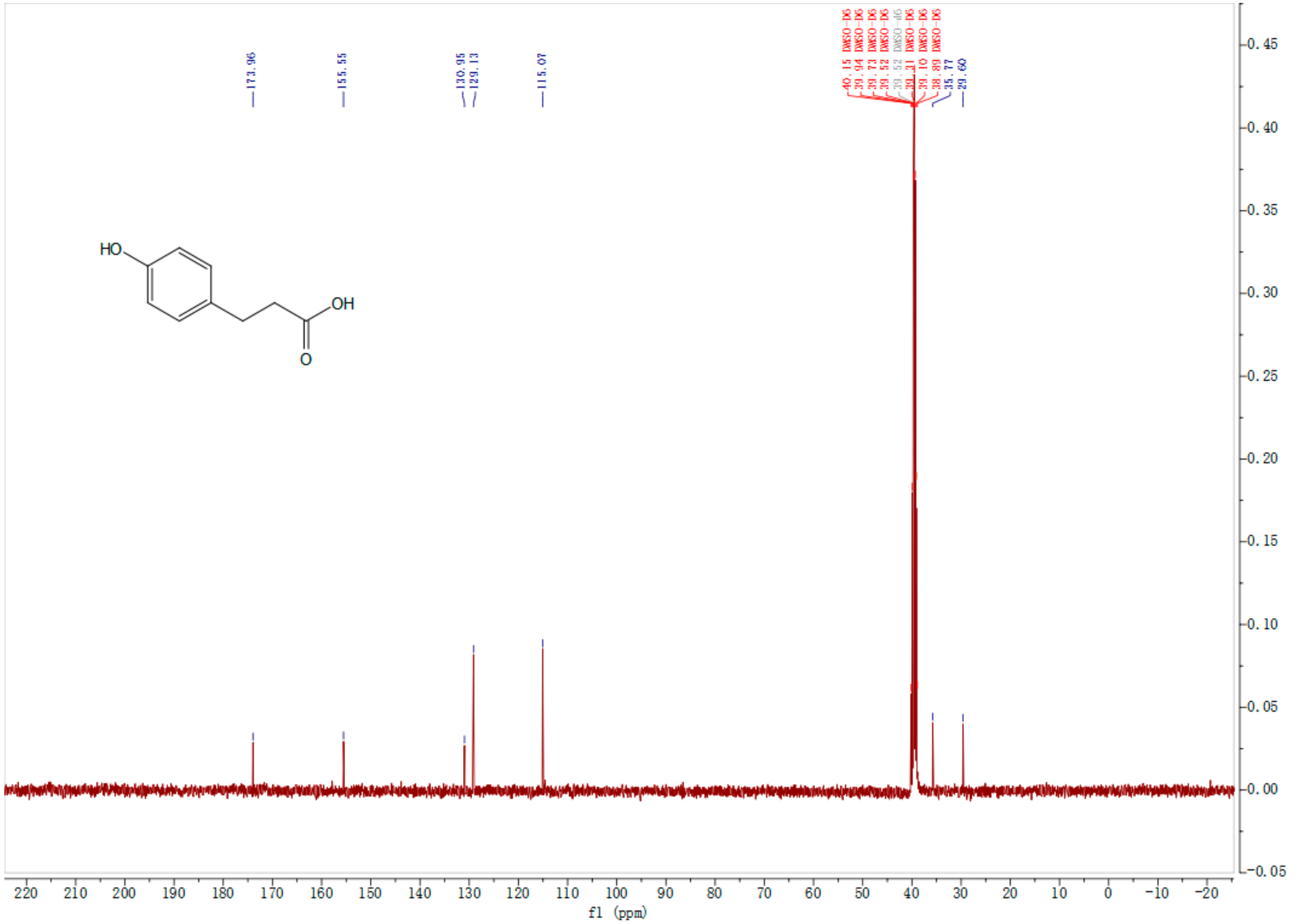

Figure S6. ^{1}H NMR of compound **3**

Ferulic acid

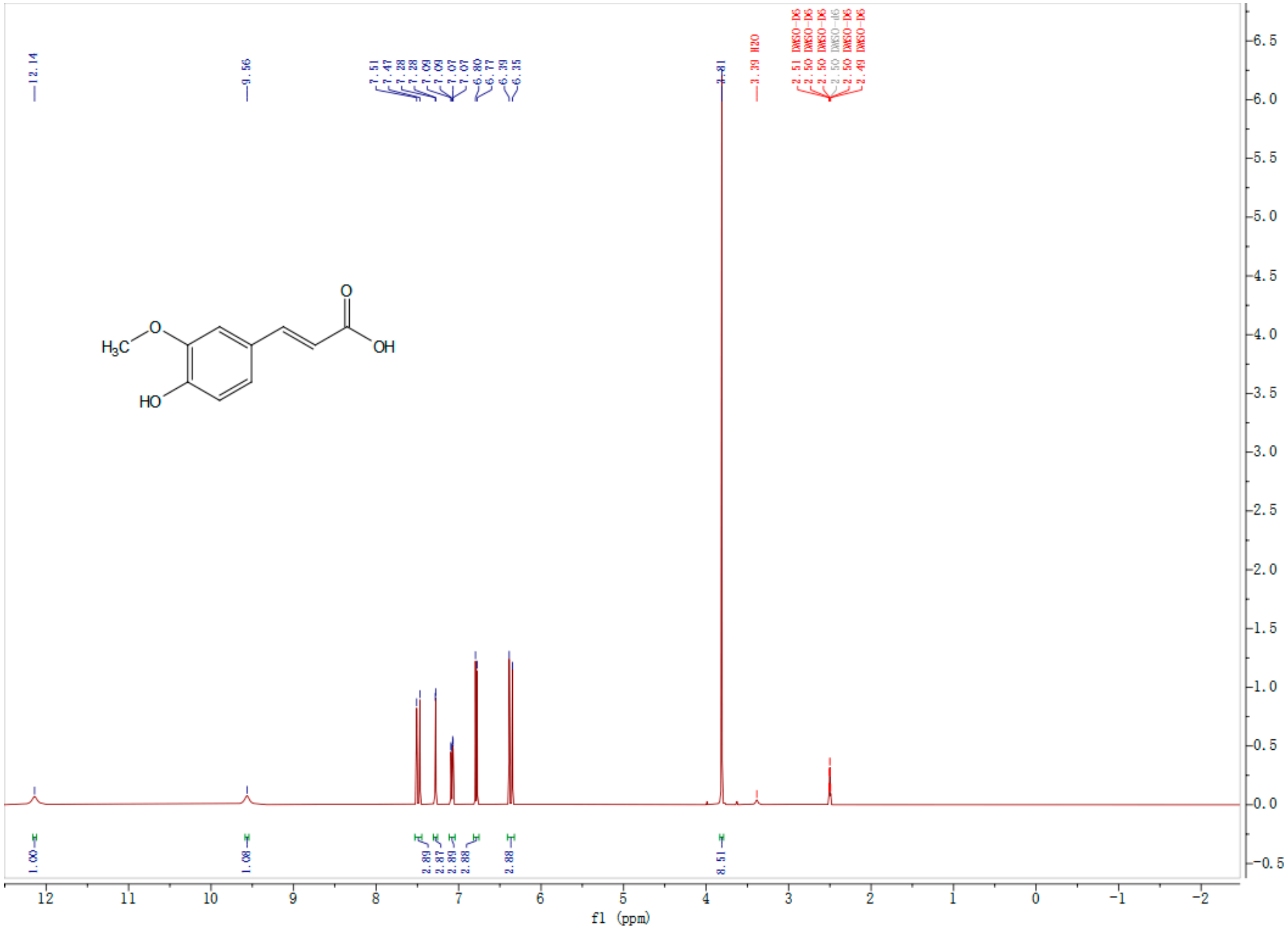

Figure S7. ^{13}C NMR of compound **3**

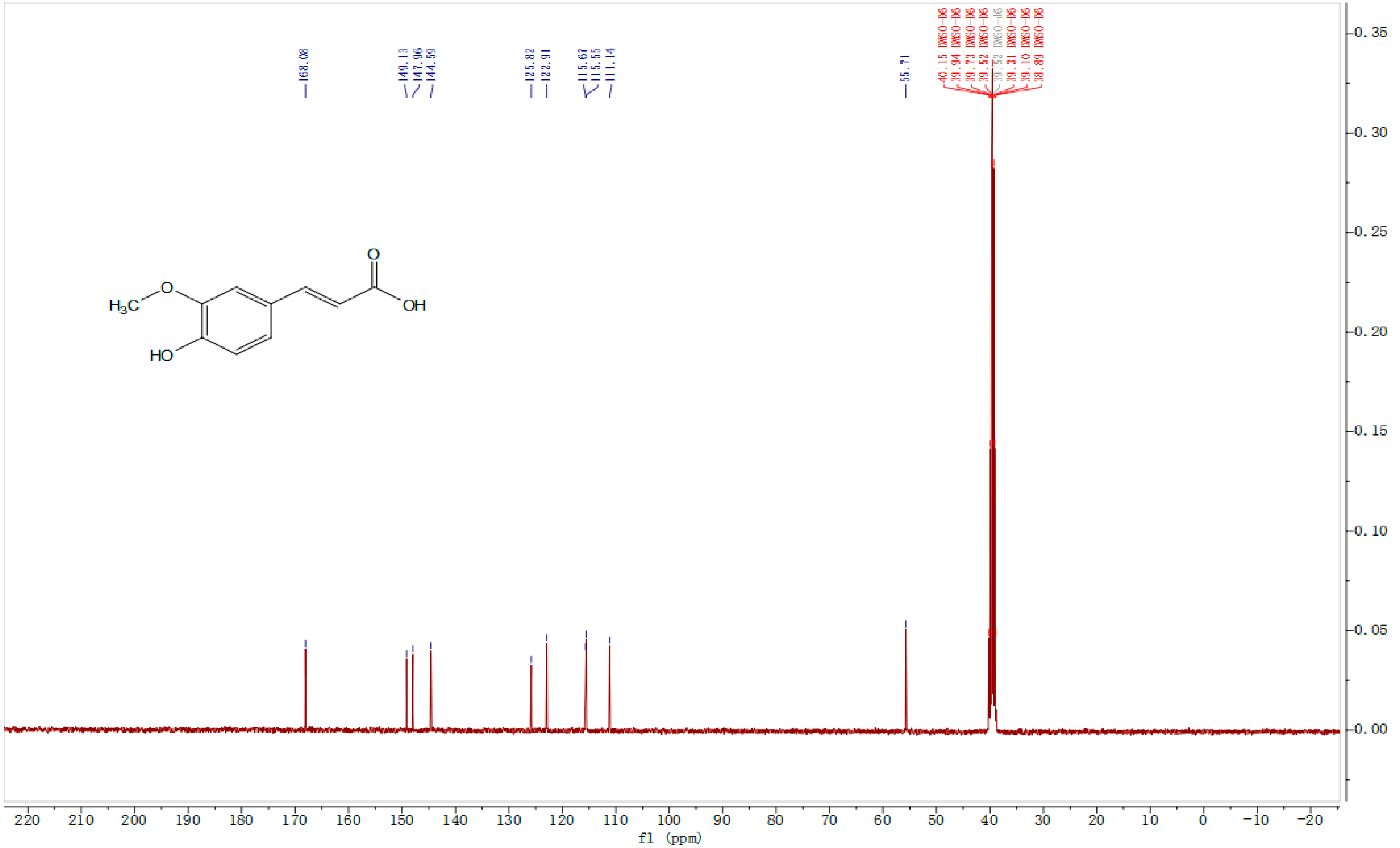

Figure S8. ^{1}H NMR of compound **4**

Maculosin

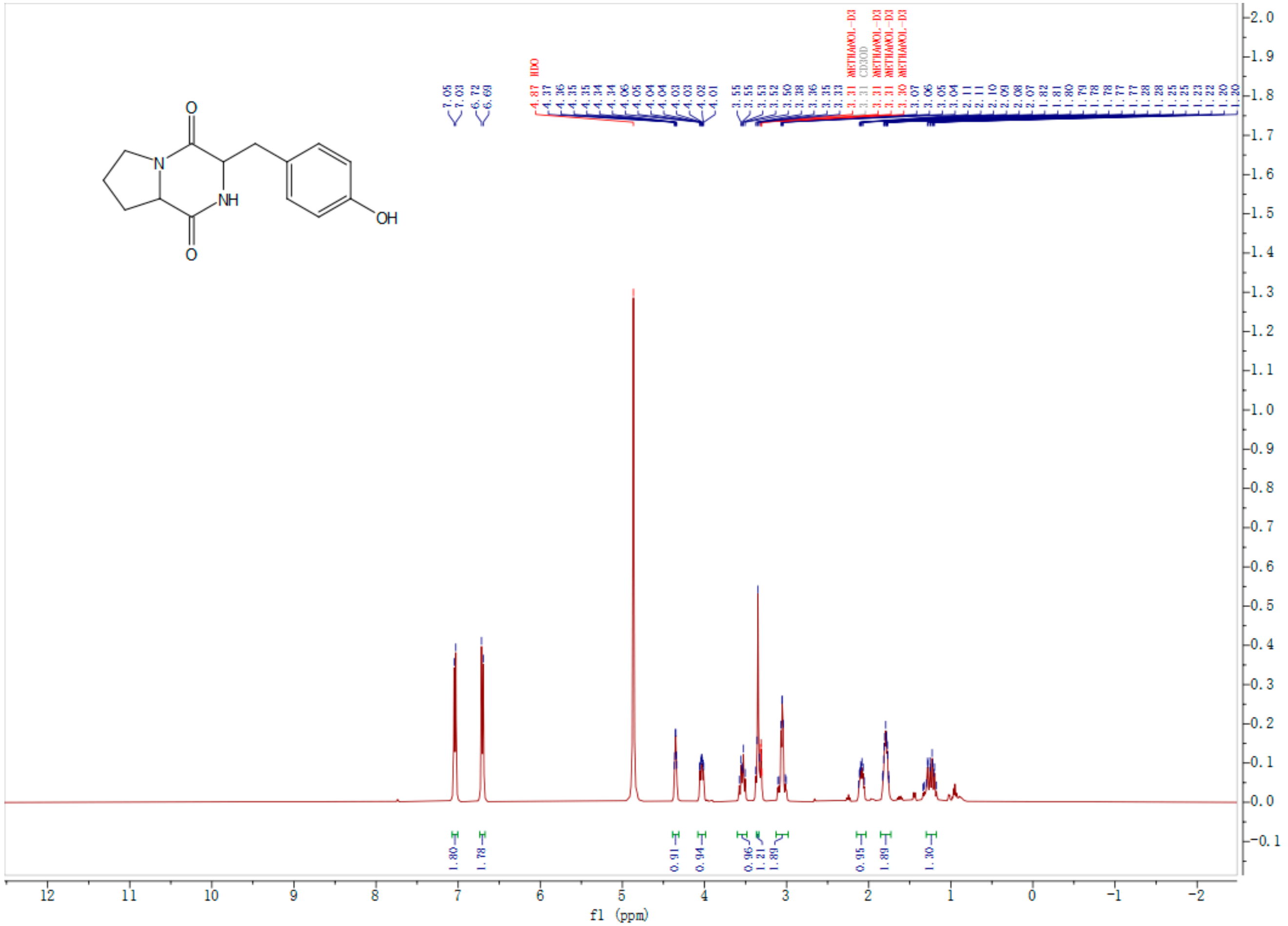

Figure S9. ^{13}C NMR of compound **4**

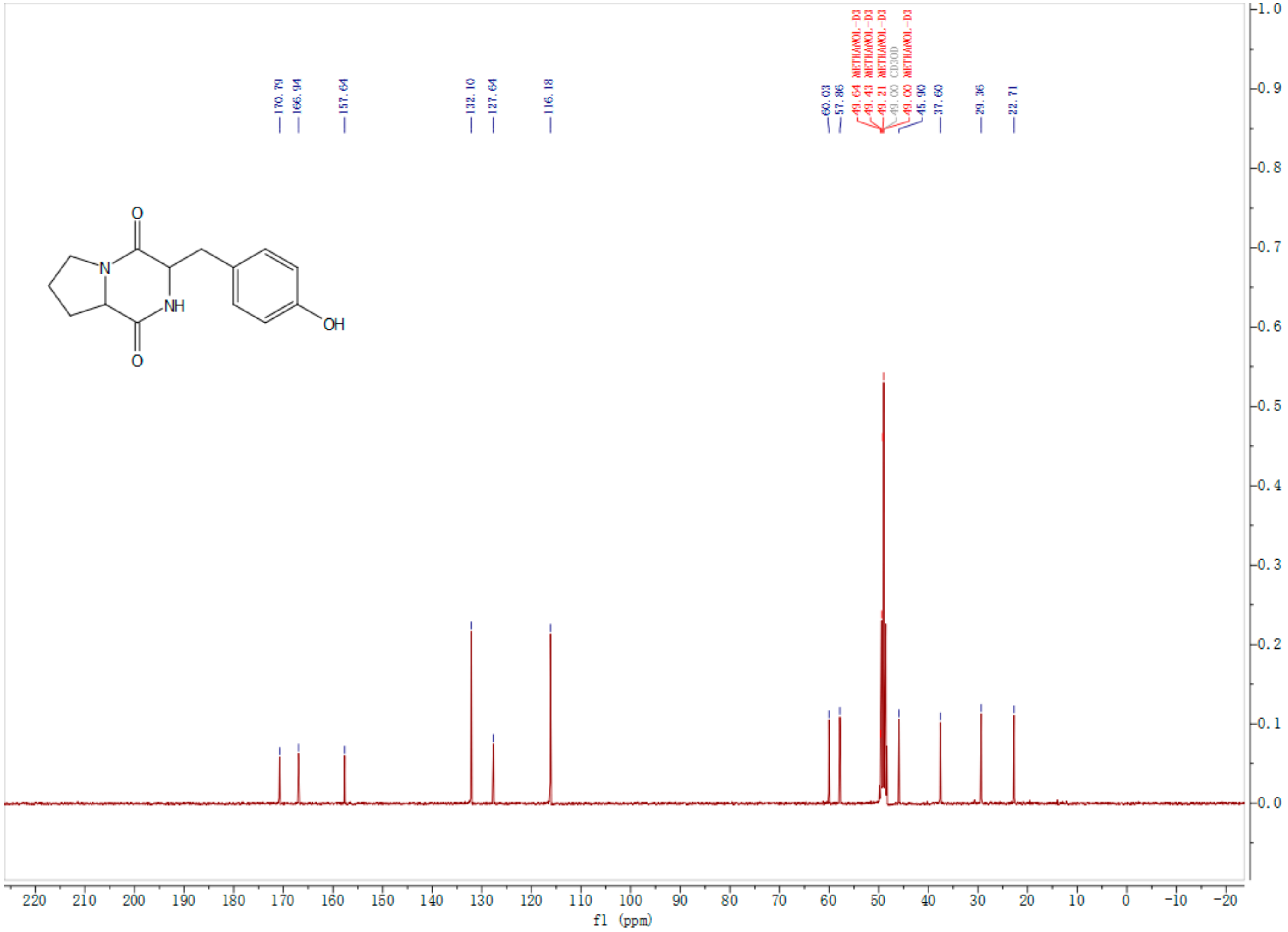

Figure S10. ^{1}H NMR of compound **5**

Brevianamide F

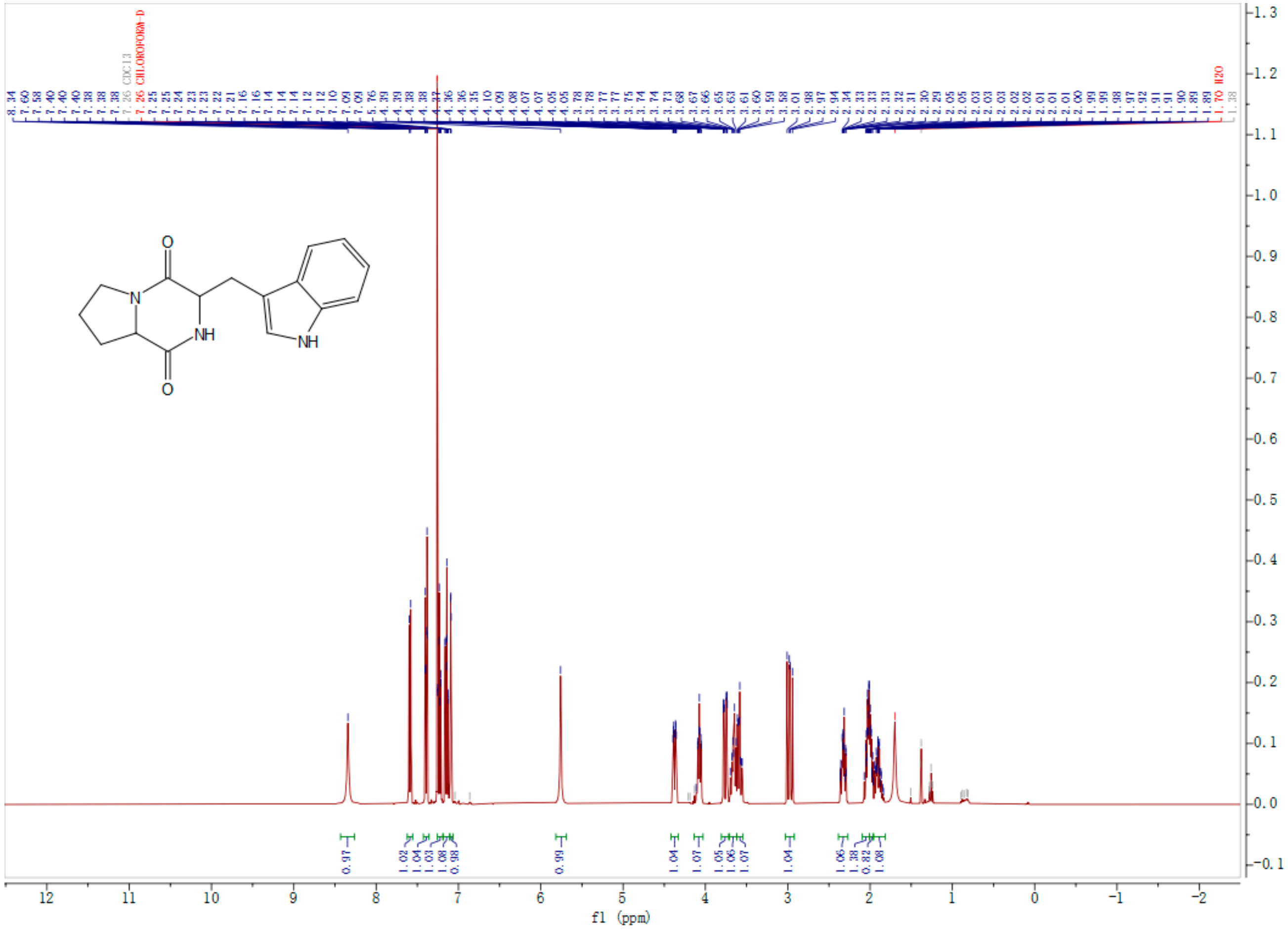

Figure S11. ^{13}C NMR of compound **5**

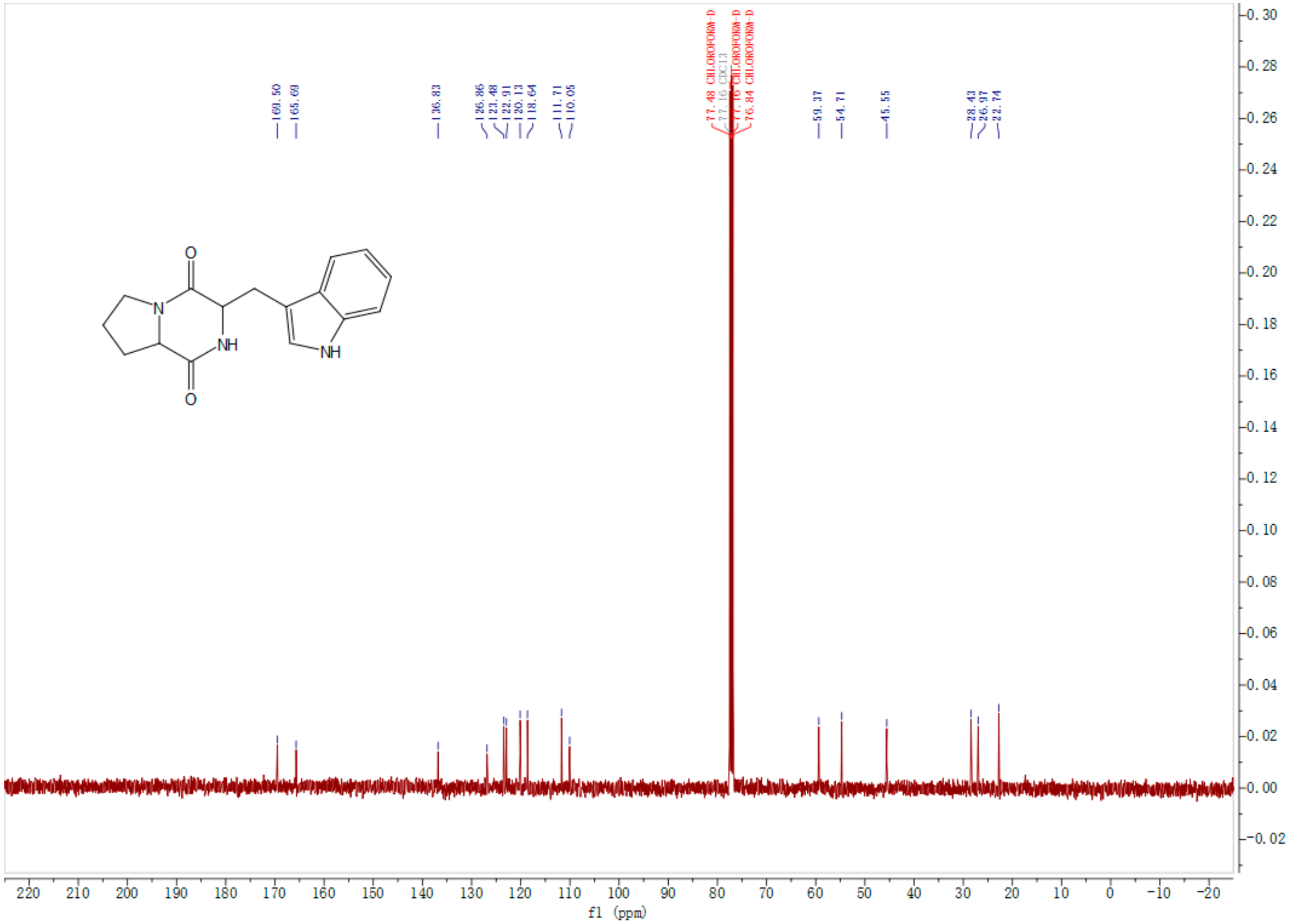

Figure S12. ^{1}H NMR of compound **6**

Indole-3-acetic acid

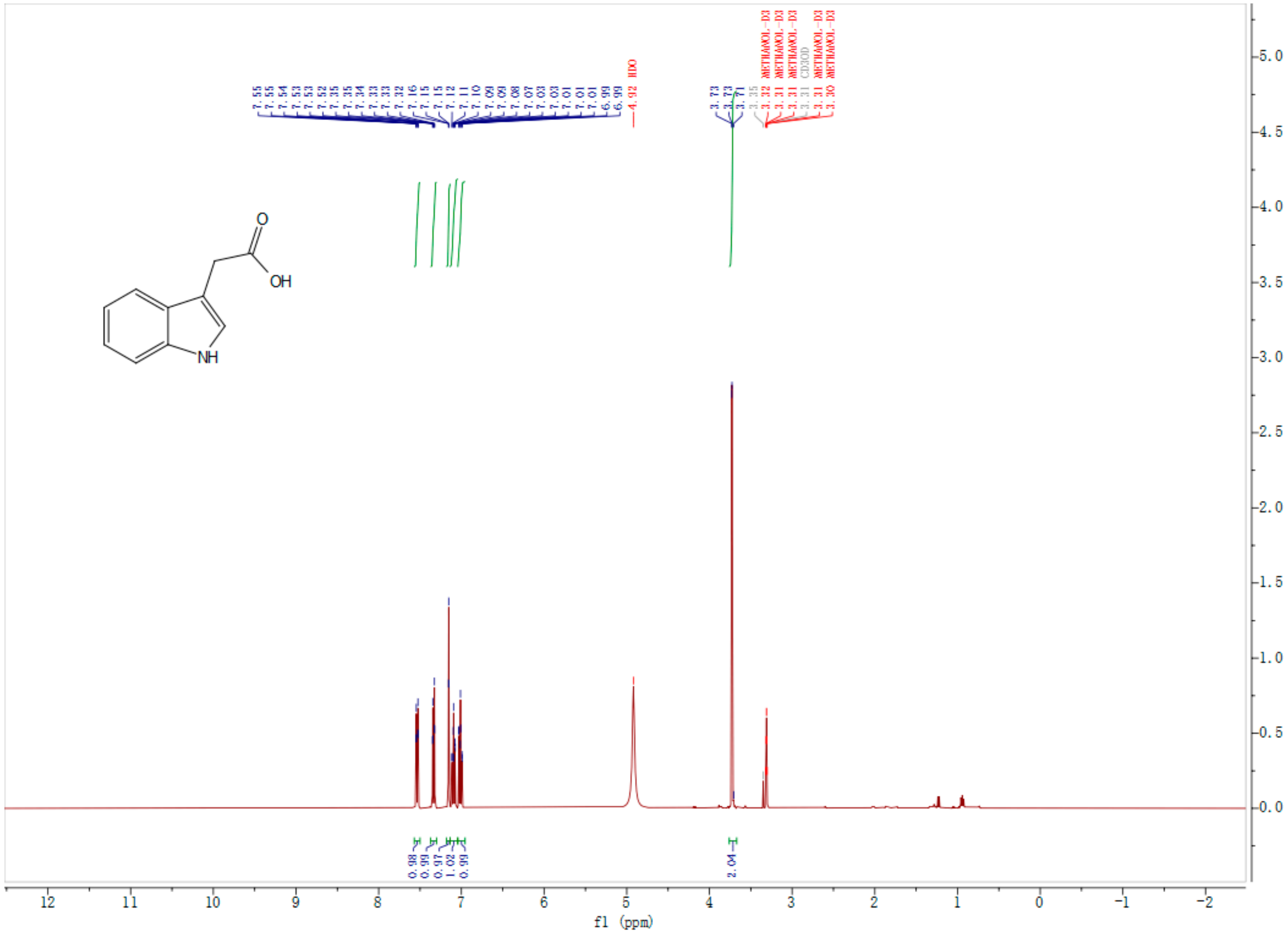

Figure S13. ^{13}C NMR of compound **6**

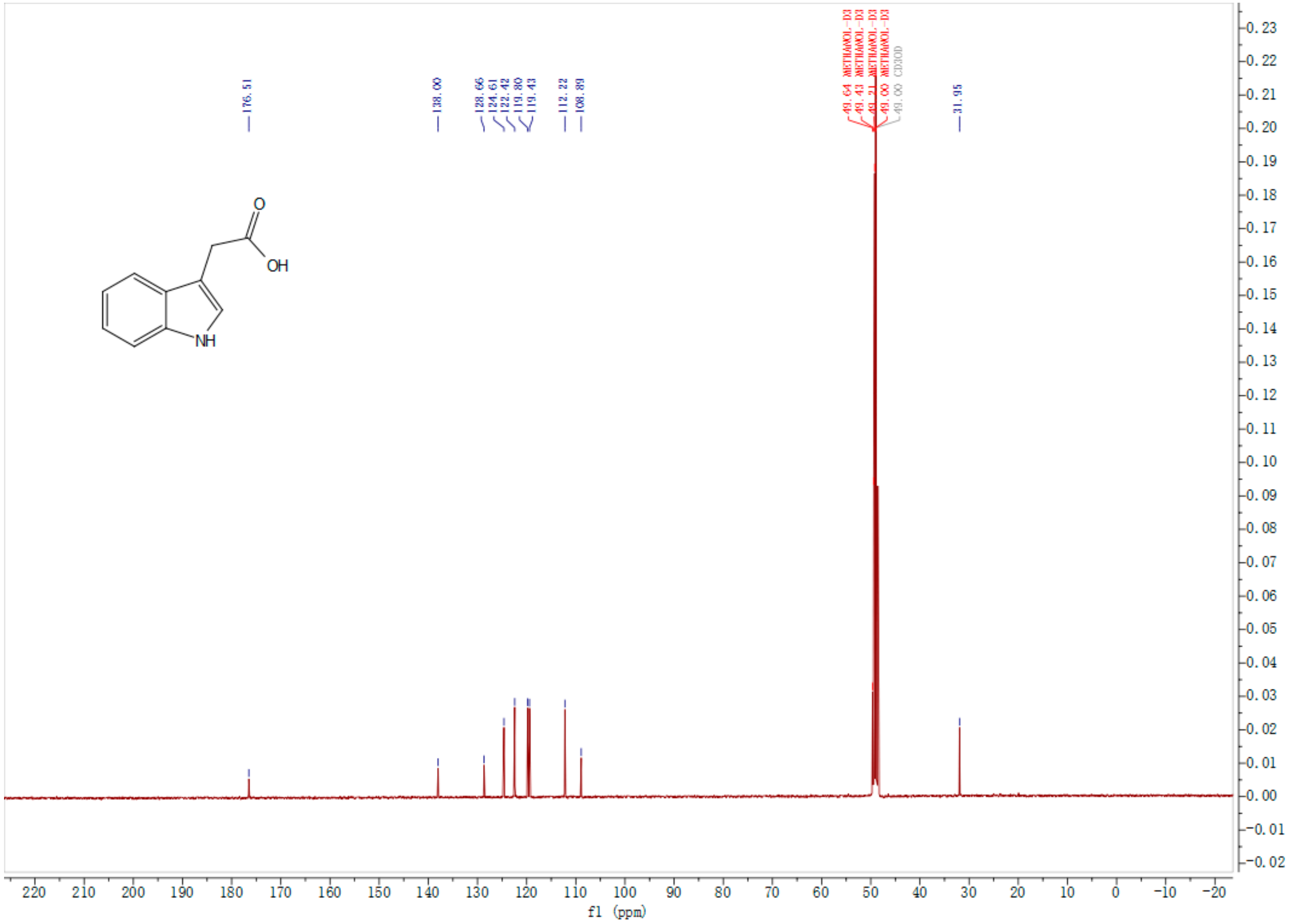

Figure S14. ^{1}H NMR of compound **7**

Butyric acid

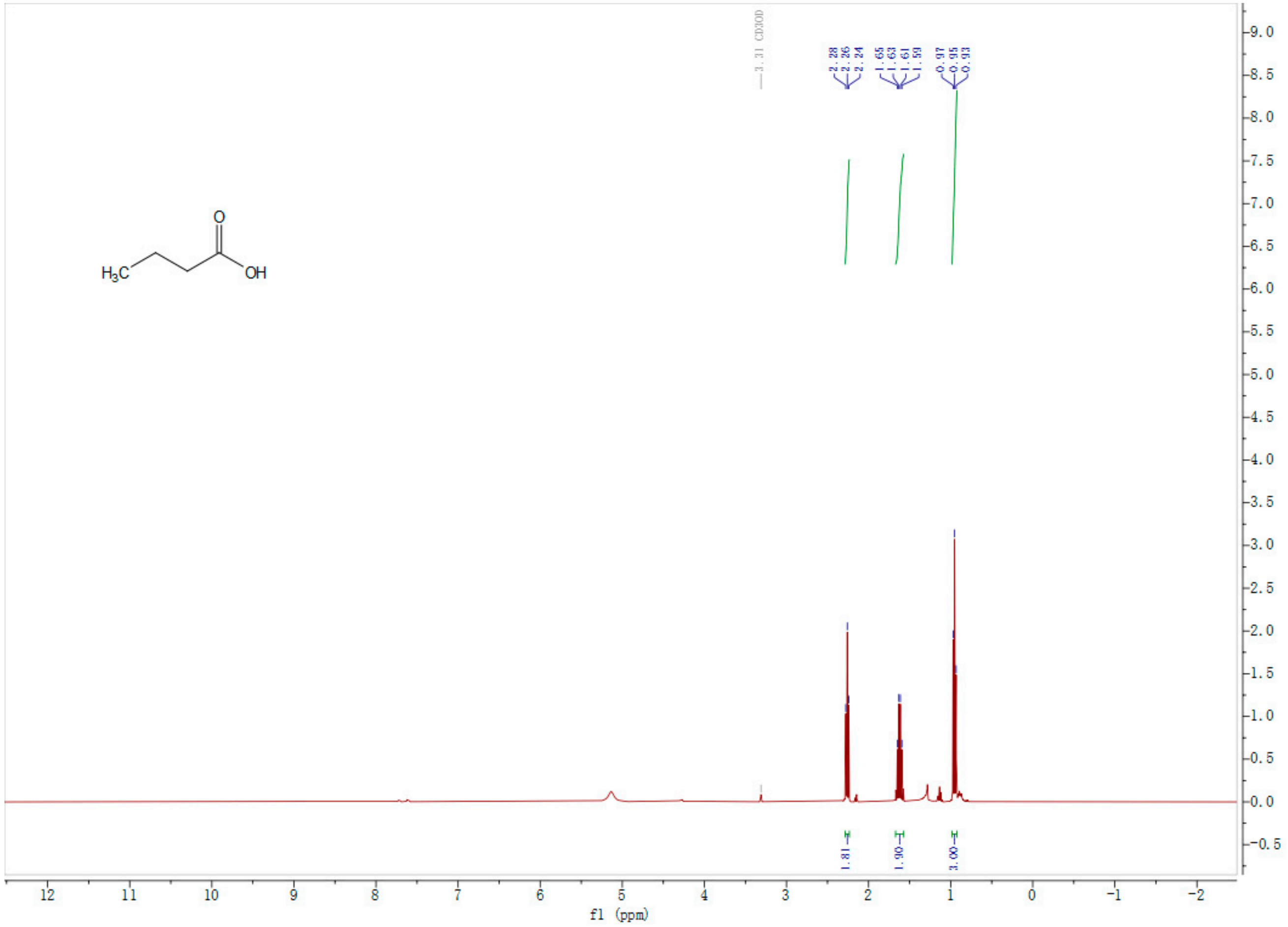

Figure S15. ^{13}C NMR of compound **7**

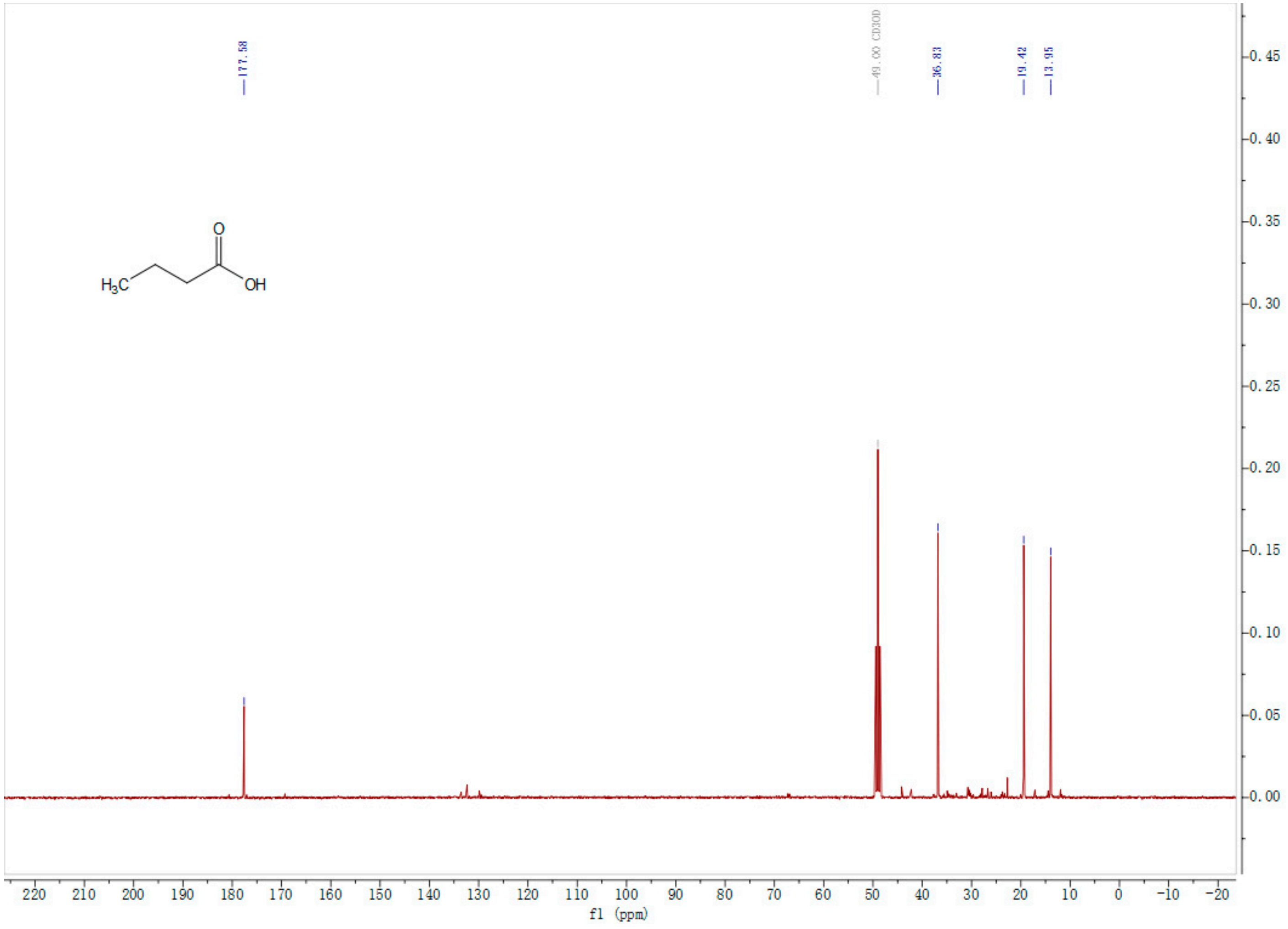

Table S1. The ADMET properties of the compounds.

ADMET properties	Tryptophol	Tropolone	Kojic acid	Arbutin
Ames mutagenicity	0.66	0.81	0.77	0.81
Acute oral toxicity	0.6983	0.6422	0.619	0.6959
Caco-2 permeability	0.9205	0.9389	0.5133	0.7795
CYP1A2 inhibition	0.6802	0.8122	0.9046	0.9469
CYP2C19 inhibition	0.5736	0.8586	0.9025	0.8964
CYP2C9 inhibition	0.5172	0.9629	0.907	0.8842
CYP2C9 substrate	0.8129	1	0.8	1
CYP3A4 inhibition	0.8848	0.9603	0.909	0.9064
CYP3A4 substrate	0.6182	0.8023	0.7325	0.5643
CYP2D6 substrate	0.4228	0.7597	0.847	0.8139
CYP2D6 inhibition	0.6408	0.9373	0.9557	0.9361
CYP2C8 inhibition	0.8111	0.9651	0.9807	0.6568
CYP inhibitory promiscuity	0.7243	0.9225	0.7747	0.7203
Human intestinal absorption	0.9964	0.9965	0.9139	0.8286
P-gp inhibitor	0.9907	0.9865	0.979	0.9527
P-gp substrate	0.9394	0.9965	0.9904	0.9829
Skin irritation	0.6766	0.9432	0.5544	0.8321
Skin sensitisation	0.7992	0.9507	0.5367	0.8479
Skin corrosion	0.8569	0.9671	0.9403	0.9721
